# Tick‐Tock, the Time Has Come: Leveraging TikTok to Understand, Prevent, and Treat Eating Disorders

**DOI:** 10.1002/eat.70091

**Published:** 2026-04-02

**Authors:** Macarena Kruger, Isabella Pruscino, Caroline G. Martin, Elizabeth A. Velkoff

**Affiliations:** ^1^ Department of Psychological and Brain Sciences Drexel University Philadelphia Pennsylvania USA; ^2^ Center for Weight, Eating, and Lifestyle Sciences Drexel University Philadelphia Pennsylvania USA; ^3^ Center for Healthcare Delivery Science Nemours Children's Health Wilmington Delaware USA; ^4^ Department of Psychology University of Toledo Toledo Ohio USA; ^5^ Department of Psychology Reed College Portland Oregon USA

**Keywords:** brief interventions, eating disorders, prevention, rapid review, short‐video format, social media, TikTok

## Abstract

**Objective:**

TikTok—a highly engaging social media platform with a powerful algorithm that displays short videos—has become massively popular in recent years. As research highlights the concerning relationship between image‐based content on social media and disordered eating symptoms, TikTok may serve as an optimal platform to understand eating disorders (EDs) and body image‐related concerns.

**Method:**

We conducted a rapid review of the research on TikTok, EDs, and body image. From an initial pool of 205 articles, 58 met inclusion criteria for the review. Research included content analyses (*n* = 19), observational studies (*n* = 23 studies reported in 22 articles), and experimental studies (*n* = 22 studies reported in 17 articles).

**Results:**

The research identified both potentially harmful content and ED recovery content present on TikTok. There are potentially positive effects of body positivity and body neutrality TikTok content on body image and ED risk.

**Discussion:**

The literature mostly includes non‐representative samples of young women. It remains unclear what effects identified in research so far are specific to TikTok, versus generalizable to short‐form video content. We conclude by discussing TikTok's potential as a platform for disseminating evidence‐based ED information and delivering brief interventions, drawing on harm reduction principles to promote TikTok as a space where providers can meet social media users with EDs where they are. Instead of encouraging users to stop their TikTok usage, we suggest that future research explore how TikTok can be leveraged as a tool for ED treatment, a crucial avenue given the limited accessibility of ED treatment.

## Summary

- We conducted a rapid review to examine the state of the literature on TikTok use, EDs, and body image.
- We found studies suggesting mixed results regarding the extent to which TikTok has a negative impact on EDs and body image‐related concerns among TikTok users.
- We recommend a harm‐reduction approach to leverage the use of TikTok to better understand EDs, disseminate ED evidence‐based information, and deliver ED brief interventions.

## 1 Introduction

The relationship between social media use and eating disorders (EDs) is established through two decades of research (e.g., Griffiths et al. 2018; Rodgers and Melioli 2016). Social media use can increase appearance comparisons, body dissatisfaction, and disordered eating (Cavazos‐Rehg et al. 2019; Griffiths et al. 2018; Holland and Tiggemann 2017; Meier and Gray 2014; Rodgers and Melioli 2016). Social media has evolved from primarily text‐based forms (e.g., Facebook, Twitter) to photo‐based platforms (e.g., Instagram), and most recently to video, as in YouTube and TikTok. TikTok, a short‐form video app, is one of the most widely used social media platforms in the world (Jung et al. 2025). TikTok introduced two innovations dramatically impacting the user experience: the emphasis on short videos and the algorithmically determined “For You Page” (FYP), which provides a continuous stream of videos (e.g., see Boeker and Urman 2022; Zannettou et al. 2024). After a legal battle in the United States amid concerns about users' data privacy, TikTok is likely to remain accessible in the US, and has influenced a shift toward short‐form videos on other platforms (McCabe and Lindner 2026).

While on most social media platforms, a user exerts control over the content they view by choosing accounts to follow, the user's experience of TikTok is heavily dictated by the algorithm. As a user scrolls their FYP, the algorithm pushes content based on characteristics of their engagement (Gabor 2023). The proprietary algorithm is described as a “black box,” and TikTok exhibits low transparency about how the algorithm selects content to feed to users (Mousavi et al. 2024; Smith 2021). Creators attempt to push their videos into others' FYP with hashtags identifying the type of content. TikTok shadowbans (i.e., blocking or partially blocking) videos that promote, glorify, or normalize EDs (Benzel et al. 2025). However, users circumvent shadowbans through modified language (i.e., *algospeak*), such as intentionally misspelling words (e.g., #anorexiaa, #thynsporation; Benzel et al. 2025; Steen et al. 2023). In addition to responding to hashtags, the algorithm is sensitive to user actions and can learn users' interests (and potentially, vulnerabilities) faster than other social media platforms, with the purpose of increasing the time that users spend on the app (Gabor 2023).

However, TikTok may also raise awareness and foster community (Greene et al. 2023; Herrick et al. 2021), as individuals with EDs express struggles and share their recovery process on social media (Mitra et al. 2023; Wenig and Janetzke 2022). Nonetheless, it remains unclear what moderators may influence the impact of #EDrecovery or other ostensibly recovery‐oriented content, and under what conditions it may be helpful versus harmful. To illustrate the potential dangers that users assume when they log on, a recent case study describes an adolescent girl whose FYP was filled with “anti‐pro‐ana” content, videos purportedly aimed at combating anorexia and raising awareness, but which she reported inspired her restriction (Logrieco et al. 2021).

Research to understand the relationship between TikTok and EDs is nascent. To inform researchers in the ED field, we conducted a rapid review of the research on TikTok, EDs, and body image, and synthesized the information to develop recommendations for future research.

## 2 Method

### 2.1 Search Strategy

**FIGURE 1:**
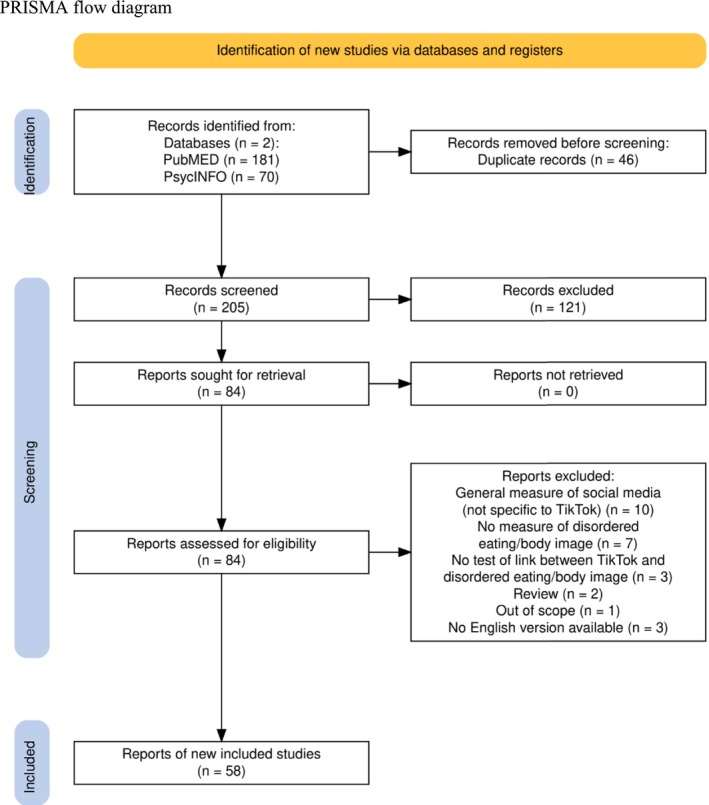
PRISMA flow diagram. PRISMA diagram depicting flow of articles through screening.

We adhered to the PRISMA guidelines (Liberati et al. 2009) for our review. On October 13, 2025, we searched PubMED and PsycINFO for the following terms: TikTok AND (“disordered eating” OR eating disorder* OR bulimia OR anorexia OR binge eating OR body* OR diet* OR weight*). Records were entered into Rayyan (Ouzzani et al. 2016), an online tool that supports systematic reviews. Likely duplicates were identified automatically by Rayyan and reviewed for accuracy by the senior author (EAV). We first screened by title and abstract; each record was screened for eligibility by all authors. Next, during full‐text screening, each record was screened by two authors. Finally, each included article was reviewed by one author for data extraction. After each stage, the review team resolved discrepancies through discussion. See Figure 1 for the PRISMA flow diagram (Haddaway et al. 2022).

### 2.2 Inclusion and Exclusion Criteria

The inclusion criteria required that articles (1) describe an empirical study, (2) be psychological research, (3) be published in peer‐reviewed journals, and (4) assess the relationship between an ED‐ or body image‐related variable and TikTok. Articles that assessed multiple forms of social media in relation to ED or body image were included only if they reported analyses isolating an effect/relationship with TikTok specifically. Articles were excluded if (1) the full text was not available in English or (2) they described case studies, systematic reviews, meta‐analyses, narrative reviews, or commentaries.

## 3 Results

The 58 included articles (Table 1) included content analyses, observational designs, and quasi‐experimental and experimental designs.

**TABLE 1:** Description of included articles.

Content analyses				
Title and authors	Content type	Sampling approach	Analytic sample	Primary TikTok‐related results
Aubrey et al. (2024) The body positive… or the body neutral? A content analysis of body positivity and body neutrality hashtagged videos on TikTok	#bodypositivitymovement, #bodypositivity, #body positive, #bodyneutralitymovement, #bodyneutrality, #bodyneutral	Systematic random sampling. After choosing a random video, every fifth video from the list on each hashtag was chosen until 66 or 67 videos were selected	394 TikTok videos	At least one body positivity theme occurred in 35.3% of videos, whereas at least one body neutrality theme occurred in 45% of videos. The most popular theme in body positivity videos was body acceptance and love (41.4% of videos), and size inclusivity (23.2% of videos) was the most popular body neutrality theme
Davis et al. (2023) A reflexive thematic analysis of #WhatIEatInADay on TikTok	#WhatIEatInADay videos	TikTok “Discover” feature identified the most‐liked videos tagged #WhatIEatInADay and used the 100 first‐appearing, relevant videos	100 TikTok videos	Two themes of #WhatIEatInADay videos emerged: Lifestyle (60% of videos) and Eating Only (40% of videos). Lifestyle videos included aesthetic elements, weight loss promotion, thin ideal, and/or ED behaviors. Eating Only videos featured highly palatable food and excessive food consumption
Greene et al. (2023) Visions of recovery: A cross‐diagnostic examination of eating disorder pro‐recovery communities on TikTok	#bedrecovery, #anarecovery, #miarecovery, #orthorexiarecovery, #arfidrecovery	Identified one related hashtag that had the most views based on searching TikTok and used Apify's TikTok scraper to collect the post URLs and captions along with metadata and relevant information about the poster	241 TikTok videos	Five themes were identified: centrality of food to EDs and recovery (69.39% of videos), what EDs look and feel like (55.60% of videos), recovery as a process (80.50% of videos), getting and giving help (50.62% of videos), and negotiating diet culture in recovery (44.40% of videos). There were statistically significant differences between ED recovery hashtags. For example, there were statistically significant differences in the presence of diet culture promotion across diagnostic hashtags [*χ* ^2^ (4, 241) = 50.34, *p* < 0.001]. #bedrecovery posts were statistically significantly more likely to include diet culture promotion than would be expected by chance (*Z* = 7, *p* < 0.001), whereas #orthorexiarecovery #arfidrecovery posts were less likely to include diet culture promotion (*Z* = −2.3, *p* = 0.021 for both). Regarding user engagement, #anarecovery posts received the most plays (Mdn = 233,850)
Greene and Norling (2023) Follow to *actually* heal binge eating: A mixed methods textual content analysis of #BEDrecovery on TikTok	#BEDrecovery	Used Apify's TikTok scraper to collect data from public TikTok posts between 2021 and 2022 tagged with #BEDrecovery	1074 post captions	Six themes emerged: (1) diets and eating approaches (57.8% of posts; subthemes: Full day of eating posts [*n* = 231], food freedom [*n* = 261], restrictive dietary approaches [*n* = 177], and emotional eating [*n* = 76]); (2) help and support (30.2% of posts; subthemes: tips [*n* = 324] and helping professional [*n* = 49]); (3) mental health (22.6% of posts; *n* = 243); (4) diet culture critique (19.1% of posts; *n* = 204); (5) body monitoring (17.5% of posts; subthemes: weight status [*n* = 188] and before and after transformations [*n* = 21]), and (6) fitness (14.4% of posts; *n* = 155). The number of shares was statistically significantly lower in posts that included diet culture critique than in those that did not (*U* = 80,324, *p* = 0.028, *r* = 0.069). Body monitoring had higher plays (*U* = 16,221, *p* = 0.003, *r* = 0.091) and shares (*U* = 16,992, *p* = 0.009, *r* = 0.079) than in those without
Hallward et al. (2023) An exploration and comparison of #BodyPositivity and #BodyNeutrality content on TikTok	#BodyPositivity #BodyNeutrality	Analyzed first‐appearing 150 English TikTok videos under each hashtag	300 TikTok videos	Three common themes emerged both from #BodyPositivity and #BodyNeutrality videos: (1) Resisting societal ideologies; subtheme: Normalizing insecurities, (2) (Re)producing disordered content; subtheme: Toxic (body) positivity promotes the need for neutrality; and (3) Social critique. Perpetuation of negative body image and promotion of disordered eating behaviors or beliefs were more likely to occur in #BodyPositivity videos. #BodyNeutrality TikTok videos may provide alternative content to promote positive body image
Harriger et al. (2023) The body positivity movement is not all that positive on TikTok: A content analysis of body positive TikTok videos	#bodypositivity	First 25 videos generated by searching #bodypositivity daily over a 14‐day period	342 TikTok videos	Most videos depicted young, White women with some features that aligned with Western beauty ideals (48.5%). TikToks rarely displayed features aligned with positive body image (67.8% of videos did not include any body positive themes) and promoted unrealistic ideals but also rarely included explicit negative appearance‐focused messaging. Weight/fat stigmatizing themes were present in 1.2% of videos, and objectification occurred in 6.4% of videos. There was a statistically significant association between body positivity themes and the extent to which objectification was present (*χ* ^2^(1) = 8.22, *p* = 0.004)
Herrick et al. (2021) This is just how I cope: An inductive thematic analysis of eating disorder recovery content created and shared on TikTok using #EDrecovery	#EDrecovery	First‐appearing 150 TikToks under #EDrecovery	150 TikTok videos	Creators (21.3%) often shared different aspects of recovery, which included 4 themes: recovery victories, reality of recovery, education, and sharing positivity. Eight posts discussed challenges experienced throughout recovery, and 7 posts challenged ED misconceptions. Eight posts shared recovery victories (e.g., smashing scales), 9 posts shared positive and motivational messages intended for others struggling with ED recovery, and 28 (18.7%) communicated their inpatient experiences using a comedic approach. Twenty‐seven creators documented and challenged the foods consumed during recovery via What I Eat in a Day posts (15.3%) and facing fear foods (2.7%). Eighteen percent featured “weight gain ‘glow up,’” and 24% of posts used trendy gallows humor (e.g., “Let's confuse people who have a good relationship with food”)
Hu et al. (2023) Chinese TikTok (Douyin) challenges and body image concerns: A pilot study	A4 waist challenge, collarbone coin challenge, and spider leg challenge	Top 30 most‐viewed videos for each challenge	90 TikTok videos	Female‐appearing individuals were in 93% of the coin challenge videos, 93% of the spider leg challenge videos, and 87% of the waist challenge videos. Video subjects had a thin body type for all three challenges (spider leg challenge: 93.3%; coin challenge: 73.33%; A4 waist challenge: 70%). Mentions of exercise were in 20% of the A4 waist, 6.67% of the coin, and 3.33% of the spider leg challenge videos. Video comments had themes of self‐comparison and thin praise. Videos of the A4 waist challenge incited more negative self‐comparison in viewers compared with the other video types
Kells et al. (2025) The experience of treatment for eating disorders as told by content creators on TikTok	#EDtreatment	100 most‐viewed videos under #EDtreatment; 45 excluded after full review due to no relevance to treatment	55 TikTok videos	The 55 videos had over 9,548,600 views, 1.8 million “likes,” 25,200 comments, and were shared with other TikTok users 18,077 times. Out of 33 total creators, 8 (24.2%) disclosed an anorexia nervosa diagnosis. Themes included aspects of treatment (69% of videos), interpersonal relationships (50% of videos), emotions and psychiatric comorbidities (38% of videos), and ED experiences (42% of videos)
Lookingbill et al. (2023) Assessment of accuracy, user engagement, and themes of eating disorder content in social media short videos	#ed, #edtiktokk, #edtiktok, #starving, #thinspho, #edrecovery, #edawareness, #edrec0very, #edawarness, #prorecovery	Stratified sampling. Downloaded 20 TikToks from each hashtag on an account created for the study	200 TikTok videos	In total, 62% of videos covered pro‐recovery content, 29.5% featured pro‐ED content, and 8.5% contained anti‐ED content. Themes included: encouraging the development or sustainment of EDs, sharing physical or emotional experiences with EDs, sharing narratives of recovery, and social support. Pro‐recovery TikToks featured more informative content than pro‐ED or anti‐ED content, (*χ* ^2^_4_ = 157.92; *p* < 0.001). There were no statistically significant differences in user engagement between informative and misleading content (likes: *F* = 0.110; *p* = 0.95; comments: *F* = 2.031; *p* = 0.13; views: *F* = 0.534; *p* = 0.59; shares: *F* = 0.691; *p* = 0.50)
Mancin et al. (2024) Let's talk about body neutrality: Content analysis of #bodyneutrality on TikTok	#bodyneutrality	Authors created two new accounts and downloaded the first 300 videos presented for potential inclusion	178 TikTok videos	Researchers described most videos as depicting individuals who are between 20 and 29 years old, look “feminine,” and present as White/Caucasian. Themes included the normalization of diverse bodies, the rejection of appearance as fundamentally important, and the idea that body neutrality is better than body positivity
Minadeo and Pope (2022) Weight‐normative messaging predominates on TikTok‐A qualitative content analysis	#weightloss, #whatieatinaday, #diet, #plussize, #weightlossjourney, #mealprep, #bodypositivity, #fatloss, #weightlosscheck, #nutrition	The first 100 videos under each hashtag	1000 TikTok videos	Most videos were created by female‐presenting (64.6%) and White‐presenting (56.1%) individuals. Nearly 44% of videos featured weight loss content, and 20.4% explicitly showed a person's weight transformation. Less than 3% featured weight‐inclusive messaging. Thirty‐eight percent explicitly showed food and 11.9% featured active cooking. Forty‐seven percent of videos under the hashtag “nutrition” provided nutrition advice, and only 1.4% were created by registered dietitians. Themes included glorification of weight loss, positioning of food to achieve health and thinness, and lack of expert voices providing nutrition information. Most posts presented a weight‐normative view of health. Nutrition‐related content is largely weight‐normative, and most posts were created by White, female adolescents and young adults
Munro et al. (2024) Diet culture on TikTok: A descriptive content analysis	#diet, #whatieatinaday, #wieiad, #dietitian, #diettips	Top 50 most‐viewed English TikTok videos from each hashtag	250 TikTok videos	Over half of the diet‐related videos featured body checking (57%). Of the videos that represented body image, almost half represented body image negatively. Most videos (54.4%) promoted “healthy eating,” and only 6.4% displayed disordered eating behaviors. Nearly half (46%) provided dietary advice, and of those videos, most content creators claimed to be experts (64%)
Nuhn et al. (2025) Understanding fitness trends in the virtual age: A content analysis of TikTok workout videos	#workout	Created a new TikTok account and collected the first 25 #workout videos that appeared each day for 2 weeks	297 TikTok videos	Most videos featured young women (62.9%) with athletic bodies (77.5%), and 55.4% included non‐White content creators. Inner‐positivity was present in 19.4% of videos, and adaptive investment in body care was present in 6.1% of videos. Body appreciation (2.9%), body acceptance (0.5%), and fat acceptance (0.5%) were rare. Themes of EDs were absent from the coded videos. Objectification was found in 24.9% of videos. There were no statistically significant differences between total body positive themes and total appearance‐focused themes, *t*(375) = −1.85, *p* = 0.07
Pryde et al. (2024) “You started working out to get a flat stomach and a fat a$$”	#fitness, #gymtok, #fittok, #fitspo	“Snapshot” approach using the “hashtag” filter (ordered videos from most to least viewed) and selected the first 50 videos from each hashtag	200 TikTok videos	The majority of TikToks featuring people were women (78%). Women were typically depicted as having a thin body (76.4%), and men were typically depicted as being of average build (60%). Most women were depicted with little to no muscle definition (45.4%) compared to men (48% with visible muscle definition). Forty‐nine percent of videos posted by fitness influencers featured objectification, followed by non‐fitness content creators (13.4%), fitness professionals (5.4%), and fitness models (1.8%). Twenty‐two percent presented information, such as dieting and exercise instructions; however, 48% of the information presented was classified as misleading and 12% as harmful. Compared to men, videos with women were more likely to contain appearance‐related captions, *χ* ^2^ (1) = 8.80, *p* = 0.003, but not diet‐related captions, *χ* ^2^ (1) = 3.17, *p* = 0.075, or exercise‐related captions, *χ* ^2^ (1) = 3.94, *p* = 0.139. Harmful or dysfunctional themes (e.g., body shaming, ED promotion, etc.) occurred in 10.7% of captioned videos, with 55.7% depicting sexualized women, 20% depicting body shaming, and 8.6% depicting ED promotion. Body shaming was the only harmful theme to occur in video captions of men (4%). Sexualization was significantly more likely in video captions of women than men, *χ* ^2^ (1) = 9.57, *p* = 0.002. Over half of videos with captions (58.9%) contained content that promoted exercise engagement for appearance reasons. Videos of women were significantly more likely to promote appearance reasons for exercise, *χ* ^2^ (1) = 8.60, *p* = 0.003, or contain no exercise components, *χ* ^2^ (1) = 9.30, *p* = 0.002, than videos of men
Raiter et al. (2023) TikTok promotes diet culture and negative body image rhetoric: A content analysis	#HealthyLifestyle	Videos under #HealthyLifestyle screened in order of most viewed to least viewed until 250 videos were found that met inclusion criteria	250 TikTok videos	The most common messages were categorized into losing weight/fat (38%), objectification (24%), and diet/specific eating messages (16%). The least common was food guilt messages (3%). Anti‐diet and body positivity messaging were observed in 10% and 7% of videos, respectively. Fourteen percent contained contradictory messages. Most of the content in #HealthyLifestyle TikToks promotes harmful body image and diet culture ideals
Rogers et al. (2025) A content analysis of #PostpartumBody videos on TikTok	#PostpartumBody	Created a new account and gradually extracted 315 of the most popular #PostpartumBody videos over a two‐week period	315 TikTok videos	The individuals in TikTok videos were primarily white (57.7%), younger than 30 (56.7%), and a healthy weight (76.2%). Fifty‐one percent included positive body image themes, and 58.4% were coded for negative appearance‐related themes. Both positive and negative themes were featured in 24.5% of the sample. Over one‐third (35.7%) of videos encouraged weight loss, 23.8% demonstrated exercise intended to change body size, 5.2% promoted dieting, and 16.8% featured body checking behaviors. “Before and after” content represented 39.9% of the sample
Romann and Oeldorf‐Hirsch (2025) Exploring algorithmic cultivation–sensitive self‐disclosure, self‐diagnosis, and hazardous mental health communication on TikTok	ED, eating disorder, mental illness‐related topics	Created a new account in incognito mode, filtered TikTok search settings to sort by “like count,” and selected the three videos with the highest number	15 TikTok videos (15,540 comments)	Themes included (1) self‐diagnosis, (2) algorithmic clock, (3) relation through similar others, and (4) emotional contagion, suggesting that the TikTok algorithm might lead users with shared mental illness experiences/symptoms to consume specific content related to them (including EDs)
Wu et al. (2025) “Mind your figure! Watch, but don't eat”: A content analysis of eating and appearance‐related messages in eating videos on social media	Mukbang, mukbang ASMR, eating broadcast, eating ASMR, competitive eating, eating challenge, eating show, eating food, eat meals	Used targeted search focused on keywords likely to identify videos primarily featuring eating and then filtered results by relevance to select 60 videos from each platform	60 TikTok videos	Videos on TikTok (compared to YouTube and Bilibili) received statistically significantly more likes and were shared more frequently than Bilibili videos, *t*(82.43) = 3.18, *p* = 0.002 (the number of shares was not visible on YouTube videos). Sixty percent of TikTok videos portrayed overeating (defined as more than three meals worth of food). Nearly half of TikTok videos portrayed thin bodies (48.3%). TikTok showed significantly fewer videos of larger bodies (1.7%) compared to Bilibili (15%) and YouTube (16.7%)

Observational studies				
Title and authors	Sample size, sampling approach, response rate	Measures	Age	Primary TikTok‐related results
Ariana et al. (2024) Influence of TikTok on body satisfaction among Generation Z in Indonesia: Mixed methods approach	Quantitative responses: *n* = 507 Qualitative responses: *n* = 32 Convenience sampling approach. The questionnaire was disseminated on social media for 30 days	The questionnaire asked questions related to TikTok activity, social media literacy regarding TikTok, appearance motivation after watching TikTok videos, thin‐ideal internalization after watching TikTok videos, upward appearance comparison on TikTok, and body satisfaction related to TikTok use Qualitative interview asked questions regarding TikTok use and body image, physical appearance comparisons, motivation to change physical appearance, and body satisfaction	Total sample Age Range = 17–26	Quantitative results: TikTok activity was not statistically significantly associated with thin‐ideal internalization (*b* = −0.082, *p* = 0.19). TikTok activity was positively associated with upward appearance comparison (*b* = 0.469, *p* = 0.002). Social media literacy on TikTok was negatively associated with thin‐ideal internalization (*b* = −0.14, *p* = 0.001). Appearance motivation was positively related to thin‐ideal internalization (*b* = 0.261, *p* = 0.002) and upward appearance comparison (*b* = 0.28, *p* = 0.001). Upward appearance comparison was positively associated with thin‐ideal internalization (*b* = 0.534, *p* = 0.003) and negatively associated with body satisfaction (*b* = −0.204, *p* = 0.003). Thin‐ideal internalization was negatively associated with body satisfaction (*b* = −0.168, *p* = 0.006) During qualitative interviews, participants revealed that the longer they spent on TikTok, the more they would be exposed to physical appearance content that shows ideal body standards. Participants reported recognizing content that promotes the thin ideal and that this can lead to body shaming. However, participants also shared that they compare their appearance to those they consider better looking even when their social media literacy is high. Respondents said that if they watched a video that presented an ideal physical appearance, they wanted to change their appearance to be more ideal. Participants with high body satisfaction revealed that they did not want to have the same appearance as those they see in TikTok videos. Finally, 53% of respondents reported thin‐ideal internalization based on what they see on TikTok, which relates to body dissatisfaction
Auf et al. (2023) Social comparison and body image in teenage users of the TikTok app	*N* = 384 TikTok users: *n* = 343 Non‐probability convenience sampling approach. The questionnaire was disseminated on social media	The questionnaire assessed participants' TikTok use. Body image was assessed with the Body Image Scale (BIS)	TikTok users (M = 16.4, SD = 2.0)	Findings revealed a significantly lower body image score among TikTok users (Mdn = 64.0, IQR: 54.0–72.0) compared to non‐users (Mdn = 67.0, IQR: 58.0–73.0, *p* = 0.037)
Caravelli et al. (2025) Associations between TikTok facial filter use and body image variables	*N* = 340 Convenience sampling approach. Participants were recruited via San Diego University's online research platform (SONA) and through Canvas pages for undergraduate psychology courses 397 participants completed the survey, yet 52 participants did not pass two attention checks and were excluded. Five participants who identified as non‐binary were excluded from analyses because of the small sample size of this gender group	Facial filter use on TikTok was assessed using the author‐developed TikTok Filter Use Scale. Participants were asked to provide their self‐perceived daily hours of TikTok use and the amount of time spent daily on TikTok as calculated by their phone. Body image concerns were assessed using the Body Image Concern Inventory (BICI)	Total sample (M = 20.3, SD = 2.68). Age Range = 18–41	Those who use appearance‐improving filters and goofy filters on TikTok reported higher levels of body image concerns (*β* = 0.38, *p* < 0.001; *β* = 0.27, *p* < 0.001). However, when both types of filters were entered into the same model, goofy filter use was no longer associated with body image concerns (*β* = 0.05, *p* = 0.49)
Dahlgren et al. (2024) Further evidence of the association between social media use, eating disorder pathology and appearance ideals and pressure: A cross‐sectional study in Norwegian adolescents	*N* = 1558 Convenience sampling approach. Twenty schools from five of Norway's counties were invited to participate, with a significant concentration in Oslo and Viken. A link was distributed to students via their school email accounts or via the schools' digital platforms	Participants were asked to respond yes/no if they use a particular social media platform (including TikTok). If yes, they were asked to indicate how long they spend on TikTok and whether the platform has had a positive or negative influence on how they feel about their appearance. The Eating Disorder Examination Questionnaire short (EDE‐QS) was used to assess eating pathology. To assess the internalization of appearance ideals, authors used the Sociocultural Attitudes Toward Appearance Questionnaire‐4 (SATAQ‐4)	Total sample (M = 17.0, SD = 0.95). Age Range = 16–23	Adolescent TikTok users reported that TikTok had a negative influence on how they felt about their appearance, especially among girls (55.1%) compared to boys (12.6%). A smaller percentage of girls (39.6%) reported that TikTok had a positive influence on how they felt about their appearance. A higher percentage of boys reported that TikTok had a positive influence on how they felt about their appearance (22.3%). TikTok users who are girls and said that TikTok had a negative influence on their appearance had increased disordered eating (EDE‐QS M = 11.2, SD = 7.9) compared to those who said that TikTok did not have a negative influence on their appearance (EDE‐QS M = 7.5, SD = 6.9, *p* < 0.001). TikTok users who are boys and said that TikTok had a negative influence on their appearance had increased disordered eating (EDE‐QS M = 5.7, SD = 4.9) compared to those that said that TikTok did not have a negative influence on their appearance (EDE‐QS M = 3.3, SD = 4.2, *p* < 0.001). TikTok users who said that TikTok had a negative influence on their appearance had significantly higher internalization of appearance ideals compared to those who said that TikTok did not have a negative influence on their appearance (across all subscales for boys and girls; *p* < 0.05)
Dajches et al. (2024) “I made you look”… and comment: Exploring the role of TikTok on body image and acceptance of cosmetic surgery	*N* = 424 Convenience sampling approach. Data were collected via a participant pool at a large southwestern university, Reddit, and Prolific 533 participants completed the survey, but 109 did not use TikTok and were removed from analyses	Appearance‐related comments on TikTok were assessed with the question “On average, how many comments do you post on TikTok videos about appearance in a typical week?” Following celebrities on TikTok was assessed with the question “How many celebrities (e.g., models, singers, actors, reality TV stars) do you follow on TikTok?” Following health/fitness accounts was assessed with the question, “How many Health and Fitness accounts (e.g., fitness bloggers, diet plans) do you follow on TikTok?” Body appreciation was assessed using the Body Appreciation Scale‐2 (BAS‐2). Body dissatisfaction was assessed using the Body Image Ideals Questionnaire (BIC). Acceptance of cosmetic surgery was assessed using the Acceptance of Cosmetic Surgery (ACSS) 15‐item scale	Total sample (M = 22.53, SD = 3.31). Age Range = 18–32	Women who post appearance‐related comments on TikTok videos have statistically significantly higher body appreciation scores (*β* = 0.112, SE = 0.440, *p* = 0.031) and statistically significant higher levels of acceptance of cosmetic surgery (*β* = 0.198, SE = 0.268, *p* < 0.001). Following celebrities on TikTok was positively associated with body dissatisfaction scores, which was further related to higher levels of acceptance of cosmetic surgery (*β* = 0.020, SE = 0.011, 90% CI [0.01, 0.05]). Following health/fitness accounts on TikTok was also positively associated with acceptance of cosmetic surgery (*β* = 0.152, SE = 0.050, *p* = 0.003)
Dondzilo et al. (2024) Association between engagement with appearance and eating related TikTok content and eating disorder symptoms via recommended content and appearance comparisons	*N* = 230 Convenience sampling approach. Participants were recruited through the University of Western Australia's departmental platform 30 participants reported that their data should not be used in the study and were excluded	ED symptoms were assessed using the Eating Disorder Examination Questionnaire (EDE‐Q). Upward social media appearance comparisons were assessed using nine adapted items from the Upward Appearance Comparison Scale. TikTok activity was assessed by asking participants about their screen time the three weeks prior. Participants accessed their watch history and reported whether each of the last 20 TikTok videos that were recommended to them contained appearance or eating content. Participants rated content as attractive	Total sample (M = 19.07, SD = 1.87). Age Range = 17–30	Engagement with appearance/eating‐related content was associated with receiving higher proportions of this content on the TikTok algorithm. This was further related to greater upward social media comparisons, which were then associated with higher ED symptoms, specifically when the content contained user‐rated appearance/eating‐related themes based on hashtags (*β* = 0.01, SE = 0.01, 95% CI [0.001, 0.031]) and when the videos contained attractive bodies (*β* = 0.04, SE = 0.01, 95% CI [0.019, 0.073])
Griffiths et al. (2024) Does TikTok contribute to eating disorders? A comparison of the TikTok algorithms belonging to individuals with eating disorders versus healthy controls	*N* = 112 Participants with EDs: *n* = 42 Healthy controls: *n* = 49 Convenience sampling approach. Participants were recruited via Australia‐based organizations and services that cater to those with EDs like the Butterfly Foundation and via advertisements delivered to psychology undergraduate students at the University of Melbourne. The study was advertised as a “study of TikTok use” 21 participants who suspected they had a current or past ED were excluded from the group‐based analyses (ED vs. healthy controls) but were retained for the analyses focused on ED symptoms	Participants provided their TikTok data. Authors scraped participant's TikTok activity and other information regarding the videos that participants came across on TikTok (e.g., hashtags and number of likes). Authors examined four categories of videos related to ED psychopathology: physical appearance, dieting, exercise, and toxic ED videos. To assess ED diagnosis, participants were asked, “In your lifetime, have you ever been diagnosed with an ED?” Those who responded “yes” were asked, “Are you currently diagnosed with an ED?” Participants' ED symptoms were further assessed with the Eating Pathology Symptom Inventory (EPSI). TikTok appearance comparisons were assessed with the Physical Appearance Comparison Scale‐Revised (PACS‐R). Authors also developed a measure for participants to self‐report on different aspects of their TikTok experience	Total sample (M = 20.63)	The algorithms of participants with EDs delivered more appearance‐oriented (adjusted for age and gender: OR = 2.46, *p* < 0.0001), dieting (adjusted for age and gender: OR = 4.35, *p* < 0.0001), exercise (adjusted for age and gender: OR = 2.42, *p* < 0.0001), and toxic ED videos (adjusted for age and gender: OR = 44.43, *p* < 0.0001) compared to healthy controls. The more severe the participant's ED symptoms were, the more likely their TikTok algorithm was biased toward appearance‐oriented (adjusted for age and gender: OR = 1.23, *p* < 0.0001), dieting (adjusted for age and gender: OR = 1.24, *p* = 0.0095), exercise (adjusted for age and gender: OR = 1.34, *p* < 0.0001), and toxic ED videos (adjusted for age and gender: OR = 2.16, *p* < 0.0001). As ED symptoms increased, the more likely participants were to “like” dieting (adjusted for age and gender: OR = 1.55, *p* < 0.0001) and toxic ED videos (OR = 1.28, *p* < 0.0001) compared to unrelated videos. Individuals with EDs reported that a higher percentage of the videos they watched contained content about diets (MD = 24.98, *p* < 0.001) compared to healthy controls. Twenty‐four participants had algorithms that delivered them over 1000 appearance‐oriented videos during the one month study period; 79% of these participants had disordered eating
Li et al. (2025) They are more beautiful than me! How social media use increases women's body‐related envy and cosmetic surgery consideration **(Study 1a)**	*N* = 257 Convenience sampling approach. Participants were recruited on the Credamo platform 260 women were recruited, but due to short response times, three were excluded from the analysis	TikTok use was assessed using a six‐item scale that asks about TikTok use intensity. Body‐related envy was assessed with the Body‐related Envy Scale (BREV) Three items were used to measure dispositional envy	Total sample (M = 31.51, SD = 7.97). Age Range = 17.75–60	A positive association was found between TikTok use and body‐related envy among women (*r* = 0.13, *p* < 0.05). Even when controlling for other variables, including dispositional envy, BMI, and income, TikTok use still predicted increased body‐related envy (*β* = 0.14, *t* = 3.48, *p* = 0.001, 95% CI [0.06, 0.22])
Li et al. (2025) They are more beautiful than me! How social media use increases women's body‐related envy and cosmetic surgery consideration **(Study 2)**	*N* = 200 Convenience sampling approach. Participants were recruited on the Credamo platform	Active and passive TikTok use was assessed using the Active and Passive Social Media Use Scale. Appearance upward comparison was assessed using the Physical Appearance Comparison Scale (PACS). Body‐related envy was assessed using the Body‐related Envy Scale (BREV). Self‐esteem was assessed with the Rosenberg Self‐esteem Scale (RSES). Three items were used to measure dispositional envy	Total sample (M = 30.55, SD = 7.58). Age Range = 18.67–57	Appearance upward comparison was positively associated with both active (*r* = 0.17, *p* < 0.01) and passive TikTok use (*r* = 0.27, *p* < 0.01). Passive TikTok use was positively associated with body‐related envy (*r* = 0.24, *p* < 0.01). Active TikTok use did not predict body‐related envy after controlling for BMI, age, income, dispositional envy, and global self‐esteem, *β* = 0.07, *t* = 1.08, *p* = 0.28, 95% CI (−0.05, 0.18). Passive TikTok use did predict body‐related envy after controlling for these variables, *β* = 0.16, *t* = 2.63, *p* = 0.009, 95% CI (0.04, 0.27). Passive TikTok use predicted appearance upward comparison, *β* = 0.25, *p* < 0.001, 95% CI (0.11, 0.38), and appearance upward comparison further predicted body‐related envy, *β* = 0.29, *p* < 0.001, 95% CI (0.18, 0.40)
López‐Gil et al. (2024) Are the use and addiction to social networks associated with disordered eating among adolescents? Findings from the EHDLA study	*N* = 653 TikTok users: *n* = 526 Simple random sampling technique. Participants were recruited in three secondary schools from the Valle de Ricote (Region of Murcia, Spain) 1378 participants completed the survey, 552 were excluded because of missing data on disordered eating, 127 participants were excluded because of missing data on social networks, and 46 participants were excluded for lack of information on other instruments	Participants described what types of social networks they use. Disordered eating was assessed with the Sick, Control, One, Fat, Food Questionnaire (SCOFF)	Total sample (M = 14.0, SD = 1.6). Age Range = 12–17	TikTok use was not related to disordered eating among adolescents (*rpb* = 0.04, *p* = 0.189)
Maes and Vandenbosch (2022) Adolescent girls' Instagram and TikTok use: Examining relations with body image‐related constructs over time using random intercept cross‐lagged panel models	*N* = 229 Convenience sampling approach. Participants were recruited from 16 schools in Flanders, Belgium, over a period of 10 months. For waves 2 and 3, the same participants were contacted by their school principals to fill out the survey at home	Respondents used a 7‐point Likert scale to indicate how long they used TikTok daily. Internalization of beauty ideals was assessed using the Sociocultural Attitudes Toward Appearance Questionnaire‐4 (SATAQ‐4). Body image self‐discrepancy was assessed using Stunkard's 9‐figure contour scale	Total sample (M = 15.12, SD = 1.50). Age Range = 12–18	In this longitudinal study, TikTok use at waves 1 and 2 did not predict internalization of beauty ideals (at wave 2: *β* = −0.10, *p* = 0.45; at wave 3: *β* = −0.17, *p* = 0.34), and internalization of beauty ideals at waves 1 and 2 did not predict TikTok use in adolescents (at wave 2: *β* = −0.06, *p* = 0.67; at wave 3: *β* = −0.01, *p* = 0.94). TikTok use at waves 1 and 2 did not predict body image self‐discrepancy (at wave 2: *β* = −0.18, *p* = 0.26; at wave 3: *β* = −0.19, *p* = 0.16), and body image self‐discrepancy at waves 1 and 2 did not predict TikTok use in adolescents (at wave 2: *β* = −0.28, *p* = 0.06; at wave 3: *β* = −0.14, *p* = 0.19)
Malhotra et al. (2025) Examining the relationship between time spent on social media platforms and body image concerns	*N* = 199 TikTok users: *n* = 82 Convenience sampling approach. Participants were recruited through the Research Experience Program Website at Swinburne University, on social media, and through participant registries held at Swinburne University 270 participants responded to the survey. 64 participants were removed as they did not complete the Eating Disorders Examination Questionnaire. One typographic error was cleaned from the data. Six participants were removed as they failed to pass ⅔ attention checks	Body dissatisfaction was assessed using the shape and weight concern subscales of the Eating Disorder Examination‐Questionnaire (EDE‐Q). Social media use was operationalized as the average number of minutes per day that participants reported spending on social media	Total sample (M = 29.9, SD = 10.55). Age Range = 18–82	Time spent on TikTok was significantly and positively associated with body dissatisfaction (*R* ^2^ = 0.093, *F*(1, 195) = 7.75, *p* = 0.042)
Mink and Szymanski (2022) TikTok use and body dissatisfaction: Examining direct, indirect, and moderated relations	*N* = 778 Convenience sampling approach. Participants were recruited through the university's Department of Psychology SONA system The initial sample was 806 participants; 13 did not finish the survey and were removed, 1 person had more than 20% missing data on at least one measure, another was under the age of 18, 4 participants failed the seriousness check, and 9 failed 2 or more attention checks	TikTok use was assessed based on two items that asked how often participants check TikTok and how long they spend on TikTok daily. Upward appearance comparisons were assessed using the 10‐item Upward Physical Appearance Comparison Scale (UPACS). Self‐objectification was assessed using the 8‐item Surveillance subscale of the Objectified Body Consciousness Scale (OBCS). Body dissatisfaction was assessed using the 9‐item Eating Disorder Inventory (EDI) Body Dissatisfaction subscale. Authors developed a scale to assess exposure to body positive media. Social media literacy was assessed using the 11‐item Social Media Literacy Scale	Total sample (M = 18.62, SD = 1.04). Age Range = 18–29	Among young adult college females, TikTok use was positively associated with upward appearance comparison (*r* = 0.16, *p* < 0.001), body surveillance (*r* = 0.17, *p* < 0.001), and body dissatisfaction (*r* = 0.14, *p* < 0.001). TikTok use was indirectly related to increased body dissatisfaction through upward appearance comparison (mean indirect [unstandardized] effect = 0.06; SE = 0.02, 95% CI [0.030, 0.093]) and upward appearance comparison and body surveillance in serial (mean indirect [unstandardized] effect = 0.02; SE = 0.01, 95% CI [0.009, 0.035]), and these relations were stronger at higher levels of acceptance and critique exposure (for upward appearance comparison only: +1 SD; *B* = 0.10; boot estimate = 0.03; 95% CI [0.055, 0.155]; for upward appearance comparison and body surveillance in serial: +1 SD; *B* = 0.03; boot estimate = 0.01; 95% CI [0.015, 0.056]) and at higher levels of commercial social media literacy (for upward appearance comparison only: +1 SD; *B* = 0.10; boot estimate = 0.02; 95% CI [0.054, 0.141]; for upward appearance comparison and body surveillance in serial: +1 SD; *B* = 0.03; boot estimate = 0.01; 95% CI [0.015, 0.051]). The interactions between TikTok use and acceptance and critique exposure (at higher levels of acceptance and critique exposure: +1 SD; *B* = 0.18, *t* = 4.47, *p* = 0.000, 95% CI [0.104, 0.266]), TikTok use and commercial social media literacy (at higher levels of commercial social media literacy: +1 SD; *B* = 0.18, *t* = 4.84, *p* = 0.000, 95% CI [0.107, 0.252]), and TikTok use and peer social media literacy (at lower levels of peer social media literacy, −1 SD; *B* = 0.17; *t* = 4.38, *p* = 0.000, 95% CI [0.095, 0.250]) predicted upward appearance comparison
O'Connor et al. (2024) Picture perfect: Exploring the relationship between problematic TikTok use, physical appearance perfectionism, and upward physical appearance comparison on body appreciation	*N* = 210 Convenience and snowball sampling approaches. Participants were recruited via online platforms 265 participants started the study: 210 consented to take part in the current study	Body image was assessed using the Body Appreciation Scale‐2 (BAS‐2). Physical appearance perfection was assessed using the Physical Appearance Perfectionism Scale (PAPS). Upward physical appearance comparison was assessed using the Upward Physical Appearance Comparison Scale (UPACS). Problematic TikTok use was assessed using the Social Media Use Questionnaire (SMUQ)	Total sample (M = 22.45, SD = 4.58)	Problematic TikTok use was associated with increased physical appearance perfectionism (*β* = 0.505, *p* < 0.001). Physical appearance perfectionism mediated the negative relationship between problematic TikTok use and body appreciation (*β* = −0.434, *p* < 0.001). No relationship was found between problematic TikTok use and upward physical appearance comparison (*β* = 0.019, *p* = 0.785) or body appreciation (*β* = 0.148, *p* = 0.052). Upward physical appearance comparison did not mediate the relationship between problematic TikTok use and body appreciation (*β* = 0.000, *p* = 0.940)
Pan et al. (2023) Female users' TikTok use and body image: Active versus passive use and social comparison processes	*N* = 7750 Convenience sampling approach. Participants were recruited through TikTok	Active TikTok use was assessed using items adapted from the Facebook Use Scale. Passive TikTok use was assessed using items adapted from the Multidimensional Scale of Facebook Use. State‐level social comparison was assessed using three items adapted from the Physical Appearance Comparison Scale‐Revised (PACS‐R). The Body‐Esteem Scale for Adolescents and Adults (BESAA) was used to measure appearance‐esteem and weight‐esteem	Total sample (M = 30.41, SD = 7.69)	Female users' active use of TikTok significantly and positively predicted their appearance‐esteem (*β* = 0.06, *t*(7, 746) = 5.30, *p* < 0.001) and weight‐esteem (*β* = 0.03, *t*(7, 746) = 3.22, *p* = 0.001), whereas passive use of TikTok negatively predicted their appearance‐esteem (*β* = −0.07, *t*(7, 746) = −6.66, *p* < 0.001) and weight‐esteem (*β* = −0.03, *t*(7, 746) = −3.50, *p* < 0.001). State‐level social comparison significantly mediated the relationship between active TikTok use and appearance‐esteem (*b* = −0.0450, [CI = −0.0517 to −0.0382]) and weight‐esteem (*b* = −0.0546, [CI = −0.0635 to −0.0461]), as well as the relationship between passive TikTok use and appearance‐esteem (*b* = −0.0127, [CI = −0.0169 to −0.0086]) and weight‐esteem (*b* = −0.0157, [−0.0210 to −0.0108]; all *p* < 0.001)
Pop et al. (2022) Body‐esteem, self‐esteem and loneliness among social media young users	*N* = 427 TikTok users: *n* = 202 Convenience sampling approach. Students in two medical universities in Romania were recruited online The survey was sent to 540 medical students; 468 students returned the survey, and 41 responses were excluded	Participants were asked about their social media use, the reason they use the app, and the average time spent on the app. Body esteem was assessed using the Body Esteem Scale for Adolescents and Adults (BESAA)	Total sample (M = 21.62, SD = 2.37). Age Range = 18–30	The more time medical students spend on TikTok, the greater their body‐esteem (*r* = 0.15, *p* = 0.002)
Primo‐Simões et al. (2025) Women's appearance‐based social comparison and dysmorphic concern: The moderating role of body appreciation among social media users	*N* = 346 TikTok users: *n* = 32 Convenience sampling with a snowball method was used. Participants were recruited on social media and through email	Participants were asked what social media platforms they prefer/used most frequently. Appearance‐based social comparisons were assessed using the Social Comparison through Physical Appearance Scale (SCPAS). Body appreciation was assessed using the Body Appreciation Scale‐2 (BAS‐2). Dysmorphic concerns were assessed using the Portuguese version of the Dysmorphic Concern Questionnaire (DCQ‐P)	TikTok users (M = 21.88, SD = 4.21)	Those who prefer TikTok (all women in this study) had significantly less favorable appearance‐based social comparisons (peers: M = 54.00, SD = 17.96, *p* < 0.001; influencers: M = 48.50, SD = 15.62, *p* < 0.001), lower levels of body appreciation (M = 3.21, SD = 0.83, *p* < 0.05), and higher levels of dysmorphic concern (M = 9.19, SD = 5.56, *p* < 0.05) compared to those who prefer Facebook (peer appearance‐based social comparisons: M = 73.60, SD = 20.42; influencer appearance‐based social comparisons: M = 66.53, SD = 23.62; body appreciation: M = 3.61, SD = 0.98; dysmorphic concerns: M = 6.54, SD = 4.98) and Instagram (peer appearance‐based social comparisons: M = 67.65, SD = 16.48; influencer appearance‐based social comparisons: M = 55.66, SD = 19.42; body appreciation: M = 3.70, SD = 0.85; dysmorphic concerns: 8.39, SD = 5.35)
Pruccoli et al. (2022) The use of TikTok among children and adolescents with eating disorders: Experience in a third‐level public Italian center during the SARS‐CoV‐2 pandemic	*N* = 78 Convenience sampling approach. Participants were recruited from outpatient and inpatient services for young patients with EDs at a third level Regional Center for Feeding and Eating Disorders of the Institute of Neurological Sciences of Bologna, Italy. The authors proposed the survey to every patient who came to the center during the selected period 91 patients were delivered the questionnaire. Thirteen patients refused to be in the survey because they did not wish to disclose their personal information	Authors developed an anonymous questionnaire to survey patients. Participants responded yes/no to questions regarding their use of TikTok, the influence of TikTok on eating attitudes, and experiences of body‐shaming related to TikTok use. A 4‐point Likert scale collected data on the frequency of viewing pro‐Ana, pro‐Mia, pro‐ED recovery, and pro‐ED awareness content. A 5‐point Likert scale assessed subjective feelings of motivation related to the visualization of the pro‐Ana/pro‐Mia content. Open‐ended questions on searched hashtags were included	Total sample (M = 14.5, SD = 2.1)	There were 50 participants (64.1%) who reported that their TikTok algorithm displayed pro‐ED recovery content and 43 participants (55.1%) reported seeing pro‐Ana content on their algorithm. Participants who reported that TikTok had a negative impact on their self‐esteem were found to frequently search for diet‐related (*p* = 0.007) and pro‐ED recovery content (*p* = 0.007). Some participants reported that a positive feature of TikTok is that it holds less space for body‐shaming (*n* = 5, 6.4%), while others reported that a negative feature is that there are body‐shaming comments on TikTok (*n* = 10, 12.8%) and that many videos may promote appearance comparisons (*n* = 9, 11.5%)
Sagrera et al. (2022) Social media use and body image issues among adolescents in a vulnerable Louisiana community	*N* = 5070 TikTok users: *n* = 1697 Convenience sampling approach. Participants were recruited in Caddo Parish schools. English teachers received the survey to administer to their classes	The Step Forward Teen Advisory Committee recommended specific question topics to Step Forward staff, which resulted in a survey of 15 questions surrounding social media use and issues with body image	Total sample (M = 15.79, SD = 1.23)	Adolescent TikTok users had significantly higher body image issues (*n* = 696, 58.4%) than non‐TikTok users (*n* = 476, 39.9%; *p* < 0.001). In comparison to other social media platforms, TikTok users had the second highest odds of self‐reporting body image issues (OR = 2.01, 95% CI = 1.74–2.31, *p* < 0.001)
Salomon et al. (2025) Weight loss content on TikTok and antifat attitudes	*N* = 556 Convenience sampling approach. College students were recruited through Sona. Another sample was recruited using Prolific	Participants were asked about their consumption of weight loss content on TikTok by describing how often they see 11 common weight loss‐related video trends. Weight bias internalization was assessed with the Modified Weight Bias Internalization Scale (WBIS‐M). Antifat attitudes were assessed using the dislike subscale of the Anti‐Fat Attitudes Questionnaire (AFA)	Total sample (M = 21.7, SD = 0.81). Age Range = 18–40	Consumption of weight loss content on TikTok is associated with higher internalized weight bias, which further increased antifat attitudes (*b* = 0.05, 95% CI [0.0186, 0.0855], *p* < 0.05)
Sanzari et al. (2023) The impact of social media use on body image and disordered eating behaviors: Content matters more than duration of exposure	2022 participants: *n* = 267 TikTok users: *n* = 228 Convenience sampling approach. Participants were recruited at a large university remotely via Qualtrics using a link provided to them via email	Participants reported the types of social media they use and their time spent on social media daily. They also responded yes/no to a question asking if they seek content related to weight loss and/or body positivity/neutrality. Body image and concern about appearance were assessed with the Body Appreciation Scale (BAS) and the Fear of Negative Appearance Evaluation Scale (FNAES). Disordered eating behaviors were assessed using the Eating Disorder Examination‐Questionnaire (EDE‐Q)	2022 participants (M = 19.05, SD = 1.50)	Participants active on TikTok (35.2%, *n* = 75) were significantly more likely to report seeking content related to body positivity/neutrality compared to those not on TikTok (13.3%, *n* = 6; *X* ^2^ = 8.62, *p* = 0.004). Those exposed to this content on any social media platform had significantly lower body appreciation scores (M = 4.60, SD = 1.30; *t*(236) = 2.40, *p* = 0.02), significantly greater fears of negative appearance evaluation (M = 3.22, SD = 1.14; *t*(231) = 4.13, *p* < 0.001), and significantly increased binge eating (M = 3.54, SD = 5.53; *t*(236) = 2.40, *p* = 0.02) compared to those not engaging with this content
Sjöström et al. (2024) Helpful or Not? A qualitative study on female adolescents' experience of TikTok when recovering from anorexia nervosa	*N* = 14 Convenience sampling approach. Participants were recruited from child and adolescent psychiatry outpatient clinics in Sweden via their providers and information in waiting rooms Out of 22 interested adolescents, 14 completed the study	Interview questions included aspects of TikTok use, types of content viewed, and if TikTok use supported participants or impacted their ED symptoms	Total sample (M = 15.2). Age Range = 14–17	Four themes emerged: (1) Social connectivity: Immediate support and recovery content, (2) Algorithmic engagement: Influence and control, (3) Regulation and adaptation: Creating safer spaces, and (4) Personal agency and recovery pathways. Participants with Anorexia Nervosa (AN) reported that TikTok provides them with a sense of relief and community and inspires recovery. However, the strong algorithm can cause participants to get “stuck in loops of AN content.” Videos regarding counting calories, eating, and ideal bodies were harmful for recovery. Participants recommended that others with EDs should stay off TikTok. Participants indicated that when they were further along in their recovery, it became easier for them to tell when TikTok was harmful
Usta Ulutaş and Okan Bakır (2025) The type of social media is a greater influential factor for orthorexia nervosa than the duration: A cross‐sectional study	*N* = 892 TikTok users: *n* = 185 Convenience sampling approach. Participants were recruited at a private university in Istanbul. The study was announced on a class basis with student representatives. Faculty members were informed, students visited classrooms, and meetings were held in common areas on campus	Orthorexia nervosa was assessed using the ORTO‐11 Scale. Social media use was assessed using The Social Media Integration Use Scale (SMUIS). Participants reported the social media platforms that they use, the time spent, and their preference for nutritional information sources on social media	Total sample (M = 20.97, SD = 2.26). Age Range = 18–65	Those who prefer TikTok were significantly associated with increased orthorexia nervosa tendency (M = 26.31, SD = 5.00), compared to those who do not prefer TikTok (M = 27.74, SD = 5.28; *p* = 0.001)

Quasi experimental/Experimental designs				
Title and authors	Sample size, sampling approach, response rate, and other relevant sampling information	Measures and experimental conditions	Age	Primary TikTok‐related results
Blackburn and Hogg (2024) #ForYou? the impact of pro‐ana TikTok content on body image dissatisfaction and internalization of societal beauty standards	*N* = 273 Experimental group (*n* = 126) Control group (*n* = 147) Snowball sampling approach. Recruitment through social media, online survey sharing platforms, and word‐of‐mouth among first‐year University students Out of the 833 participants who initially consented to participate, 555 responses were incomplete for data analysis. Among complete responses, nine were excluded due to not meeting eligibility criteria, and one was excluded due to prior ED diagnosis Most participants in this study (71%) reported using TikTok up to 2 h per day, with entertainment, fashion, beauty/skincare, cooking/recipes, and life hacks/advice as the most common themes in their FYP The rationale of the study was explicitly shared with participants at the end of the study, and participants in the experimental condition were debriefed about the non‐normative nature of the videos they were exposed to	Participants were randomized into one of the two conditions: experimental (pro‐ana TikTok video) or control (“normal” TikTok video). The experimental group was exposed to 7‐ to 8‐min TikTok videos with explicit disordered eating messages (e.g., women restricting their food and sharing weight loss tips) or implicit body image ideals (e.g., fitspiration‐style content). The control group was exposed to 7‐ to 8‐min TikTok videos displaying nature scenes, cooking and recipes, animals, and comedy Participants reported their daily TikTok usage and completed the Eating Attitudes Test (EAT‐26), ORTO‐15, Body Image States Scale (BISS), and Sociocultural Attitudes Toward Appearance Questionnaire‐4 (SATAQ‐4)	Total sample Age Range = 18–28	Women in the experimental group exposed to pro‐anorexia (“pro‐ana”) content showed the greatest decrease in body satisfaction, showing a statistically significant interaction between condition and time: Wilks' Lambda = 0.98, *F*(1, 271) = 6.83, *p* = 0.009, partial eta squared = 0.03. Similarly, the experimental group also showed the greatest increase in internalization of appearance ideals compared with the control group, demonstrated through a statistically significant interaction between group condition and time after exposure; Wilks' Lambda = 0.97, *F*(1, 271) = 8.16, *p* = 0.005, partial eta squared = 0.029. Further, despite a lack of statistical significance in ORTO‐15 scores, *F*(3, 269) = 0.38, *p* = 0.78 and EAT‐26 scores, *F*(3, 269) = 1.21, *p* = 0.31 across the four TikTok usage groups, anecdotally, women with high (2–3 h) or extreme (3+ hours) daily TikTok use showed greater ED symptoms (e.g., restrictive disordered eating and “healthy” disordered eating)
Davis et al. (2025) Does watching short‐form #WhatIEatInADay videos impact eating disorder cognitions and urges to engage in eating disorder behaviors? An experimental investigation of TikTok	*N* = 481 Lifestyle (*n* = 159) Eating Only (*n* = 159) Art Control—no eating, weight, or body‐related content (*n* = 163) Convenience sampling approach. Participants were recruited using the university SONA System's undergraduate psychology student pool Deception was used to mask the purpose of the study, so participants knew they would be viewing TikTok videos but did not know about the conditions or the content that they were going to be exposed to. At the end of the study, they were debriefed and had the opportunity to provide feedback Participants in this study reported TikTok as the most used social media platform, and 97.3% of the sample reported using social media platforms at least 30 min per day, whereas 75% reported using social media between 1 and 4 h per day Out of the 485 participants who were included in the study, four incorrectly answered at least one of the attention check questions and were removed from the study	Participants were randomized into one of the three conditions and watched 6 min of TikTok videos corresponding to their assigned condition. Participants in the Lifestyle condition were exposed to 12 videos, those in the Eating Only condition were exposed to 7 videos, and those in the Art Control condition were exposed to 17 videos Participants completed the same measures pre‐ and post‐exposure. They responded to social media use questions, completed the Eating Pathology Symptoms Inventory (EPSI)—body dissatisfaction subscale, the Clinical Impairment Assessment (CIA) to assess eating pathology and impairment, and the Visual Analogue Scales (VAS) to assess preoccupation with weight/shape and urges to engage in ED behaviors	Total sample (M = 19.04, SD = 1.14). Age Range = 18–26	Across both experimental conditions, weight/shape preoccupation scores were statistically significantly higher after exposure: Lifestyle (*t*(156) = 4.74, *p* < 0.001) and Eating Only (*t*(157) = 3.19, *p* = 0.005), with a small effect size (*p* = 0.009, *η* ^2^_*p*_ = 0.02). Specifically, a significant three‐way interaction between condition, time, and gender suggested that women exposed to Lifestyle #WIEIAD TikTok videos showed increased weight/shape preoccupation (*t*(124) = 5.46, *p* < 0.001). After FDR *p*‐value correction, this result held true for men, too Even though there was not a statistically significant main effect of time (*p* > 0.05, *η* ^2^ = 0.00) on the urge to vomit, after watching Lifestyle and Eating only #WIEIAD videos, participants reported a greater urge to vomit compared with baseline levels (when binary gender was not included in the model). Participants in the control condition presented statistically significant lower urges to binge eat (*t*(158) = 2.80, *p* = 0.006) and exercise (*t*(159) = 2.57, *p* = 0.01) after viewing #Art videos. Finally, participants in the Lifestyle condition reported a statistically significant increase in urge to exercise (*t*(155) = 4.32, *p* < 0.001), whereas those in the control condition reported a statistically significant decrease in urge to exercise (*t*(159) = 2.57, *p* = 0.01)
de Brabandere et al. (2025) #Fittok: How fitfluencers' videos on Tiktok impact adolescents' body satisfaction, workout intention, and behavior **(Study 1)**	*N* = 221 Fitfluencers' TikTok posing videos (*n* = 109) Fitfluencers' TikTok workout videos (*n* = 112) Convenience sampling approach. Participants were students from a Flemish secondary school Of the 223 responses collected for this study, one was immediately removed from the study because the participant did not consent, and another one was removed because the participant was not within the required age range of the study 117 participants reported not following any fitfluencers, and among those who do, they follow five fitfluencers on average. No additional information about participants' history of TikTok use was reported Participants were asked to view videos carefully and respond to the next questions after 2 min. After completion of the study, participants were debriefed and received information about the goal of the study and mental health resources	Participants were randomized into one of the experimental conditions. Participants in both conditions watched seven TikTok videos of different fitfluencers of a range of races for 2 min Participants only completed post‐exposure measures: Intention to workout measure, State body satisfaction measure, Self‐efficacy measure, Assimilative social comparison measure, Contrastive social comparison measure, and State appearance comparison measure *Of note, information about the measures utilized was reported incompletely/inconsistently including the timepoints at which they were administered.	Total sample (M = 16.22, SD = 1.16). Age Range = 14–18	There were no statistically significant direct effects of the type of video (i.e., fitfluencers' posing or workout TikTok videos) on participants' body satisfaction (*c*′ = 0.09, *p* = 0.45) or workout intention (*c*′ = 0.06, *p* = 0.64). Three mediators were also examined: self‐efficacy, assimilative, and contrastive comparison feelings, but all of them were not statistically significant showing no indirect effects of video type on body satisfaction or workout intention. However, self‐efficacy positively impacted body satisfaction (*b*_1_ = 0.22, *p* < 0.001), whereas contrastive comparison feelings negatively impacted body satisfaction (*b*_3_ = −0.30, *p* < 0.001). Additionally, self‐efficacy (*b*_1_ = 0.35, *p* < 0.001) and assimilative comparison feelings (*b*_2_ = 0.50, *p* < 0.001) positively impacted workout intention
de Brabandere et al. (2025) #Fittok: How fitfluencers' videos on Tiktok impact adolescents' body satisfaction, workout intention, and behavior **(Study 2)**	*N* = 176 Fitfluencers' posing videos (*n* = 89) Lifehack (control) videos (*n* = 86) Not all participants completed baseline measures. They were randomized to either complete baseline body satisfaction and workout intention measures or not, which created two additional conditions: Fitfluencers' posing videos: pre‐ and post‐exposure measures (*n* = 43), post‐exposure only measures (*n* = 46). Lifehack videos (control): pre‐ and post‐exposure measures (*n* = 44), post‐exposure only measures (*n* = 42) Convenience sampling approach. Participants were students from a Flemish secondary school Of the 191 responses that were collected, two were immediately removed from the study because participants did not consent; three were removed because they were not within the required age range of the study; five were removed because they failed the attention checks; three were removed because they completed less than 50% of the questionnaire; and two were removed because they provided a monotonous answering pattern 89 participants reported following fitfluencers in social media, and they reported following four fitfluencers on average. No additional information about participants' history of TikTok use was reported Participants were asked to view videos carefully and respond to the next questions after 1.5 min. After completion of the study, participants were debriefed and received information about the goal of the study and mental health resources	Participants were randomized into one of the experimental conditions. In the experimental condition (posing videos), participants were exposed to 7 videos with the same fitfluencer to foster feelings of parasocial interaction. The control (lifehack videos) included hands of people explaining the lifehacks, allowing participants to fill out appearance comparison measures. Each participant (across conditions) was exposed to the videos for 1.5 min Participants completed an intention to workout measure, a state body satisfaction measure, an assimilative social comparison measure, a contrastive social comparison measure, a state appearance comparison measure, and an adaptation of the athletic ideal internalization measure	Total sample (M = 14.94, SD = 0.83). Age Range = 14–18	Body satisfaction was not statistically significantly different between baseline and post‐exposure regardless of the condition (i.e., TikTok lifehack videos vs. fitfluencers' posing videos; *c′* = 0.09, *p* = 0.60). Conversely, participants showed a decrease in workout intention from baseline to post‐exposure across both conditions, although it was not statistically significant (*c*′ = −0.01, *p* = 0.97). Importantly, participants who were exposed to fitfluencers' posing videos showed greater appearance comparison than those exposed to the control condition (*a*_1_ = 1.45, *p* < 0.001), which in turn decreased their body satisfaction (*b*_1_ = −0.28, *p* < 0.001). However, there was no significant indirect effect on workout intention via appearance comparison (*b*_1_ = 0.10, *p* = 0.28). Further, exposure to fitfluencers' posing videos led to greater contrastive comparison feelings compared with the control group (*a*_2_ = 1.12, *p* < 0.001). Finally, body satisfaction did not significantly differ between baseline (M = 4.20, SE = 0.12) and post‐exposure (M = 4.17, SE = 0.13); *F*(1, 85) = 0.46, *p* = 0.50, *η* ^2^_*p*_ = 0.01. Conversely, workout intention significantly decreased, *F*(1, 85) = 4.70, *p* = 0.03, *η* ^2^_*p*_ = 0.05 from baseline (M = 4.63, SE = 0.18) to post‐exposure (M = 4.45, SE = 0.19)
de Brabandere et al. (2025) #Fittok: How fitfluencers' videos on Tiktok impact adolescents' body satisfaction, workout intention, and behavior **(Study 3)**	*N* = 154 Fitfluencers' authenticity TikTok videos (*n* = 52) Fitfluencers' posing TikTok videos (*n* = 55) Lifehack (control) TikTok videos (*n* = 47) Convenience sampling approach. Participants were students from a Flemish secondary school Of the 165 responses that were collected, two were immediately removed from the study because they did not consent; two were removed because they were not within the required age range of the study; five were removed because they failed the attention checks; and two were removed because they provided a monotonous answering pattern Participants were asked to view videos carefully and respond to the next questions after 2 min. After completion of the study, participants were debriefed and received information about the goal of the study and mental health resources	Participants were randomized into one of the experimental conditions. In each condition, participants were exposed to 2 min of TikTok videos. Participants in the authenticity condition were exposed to five types of videos: workout/instructional video, challenges/setbacks video, motivation video, progress video, and exercising/working hard video. Participants in the posing and control conditions were exposed to eight videos, of which seven were identical to those in study 2 Participants completed an intention to workout measure both pre‐ and post‐ exposure, a state body satisfaction measure both at pre‐ and post‐ exposure, an assimilative social comparison measure, a contrastive social comparison measure, a self‐efficacy measure, and a state appearance comparison measure. After exposure, participants were also instructed to “do as many lunges as [they] want for a minute”	Total sample (M = 17.06, SD = 0.67). Age Range = 14–18	Across the three conditions (experimental and control conditions), there were no statistically significant changes in body satisfaction between baseline (M = 4.69, SE = 0.08) and post‐exposure (M = 4.69, SE = 0.09; *F*(1, 151) = 0.003, *p* = 0.96, *η* ^2^_*p*_ < 0.001). Even though there was a statistically significant decrease in workout intention (*F*(1, 151) = 12.96, *p* < 0.001, *η* ^2^_*p*_ = 0.08)from baseline (M = 4.85, SE = 0.13) to post‐exposure (M = 4.58, SE = 0.14); these results held true across all conditions, which indicates no differential impact on workout intention depending on fitfluencer content. Further, there were no direct effects of the type of videos (authenticity, posing, or lifehack) either on body satisfaction (*c*′*x*_1_ = 0.11, *p* = 0.27) or post‐exposure workout intention (*c′x*_1_ = −0.22, *p* = 0.19). However, there were indirect effects on body satisfaction via contrastive comparison feelings of lifehack versus posing videos (*a*_2_ *x*_1_ = 0.72, *p* = 0.002) and lifehack versus authenticity videos (*a*_2_ *x*_2_ = 0.86, *p* < 0.001)
Di Michele et al. (2023) # SexyBodyPositive: When sexualization does not undermine young women's body image **(Study 2)**	*N* = 316 Sexualized beauty ideals (*n* = 106) Sexualized body positivity (*n* = 105) Non‐sexualized body positivity (*n* = 105) Snowball sampling approach. Participants were recruited via ads on online platforms Of the 346 participants who filled out the questionnaire, 12 were removed from the study because they did not identify as women, 7 were removed because they were not within the required age range of the study, 5 were removed due to failing the attention check, and 6 participants were removed since they were too slow completing the survey As part of the cover story, participants were told that researchers were exploring short‐term memory processes of media content, and they were told that the memory task would be preceded by interfering tasks. After completion of the study, participants were debriefed and received a written explanation of the true aims of the study	Across the three conditions, participants watched 10 TikTok reels (2 min 40 s long). They were only exposed to about 10–15 s of each soundless video. Depending on the condition that participants were randomized into, videos portrayed either (1) sexualized women (e.g., women engaging in sexualized poses or dances emphasizing their breasts) conforming to the cultural beauty ideal (e.g., thin and toned); (2) women promoting body‐positivity content (e.g., overweight, skin imperfections) in a sexualized way; or (3) body‐positivity content in a non‐sexualized way Participants completed the state Visual Analogue Scale (VAS) both pre‐ and post‐exposure to conditions to assess body satisfaction and mood; the State Appearance Comparison Scale (SACS) to assess the degree of appearance social comparison; the Self‐Objectification Questionnaire (LSOQ) to assess self‐objectification and the importance given to appearance‐ over competence‐related attributes; and the Consider subscale of the Acceptance of Cosmetic Surgery Scale (ACSS) to assess participants' intention to undergo cosmetic surgery in the future	Total sample (M = 23.87, SD = 4.19). Age Range = 19–32	In this study (study 2), there was a significant condition × time interaction found (*F*(2, 313) = 20.88, *p* < 0.001, *np* ^2^ = 0.12), such that participants reported lower body satisfaction after being exposed to the sexualized beauty ideal TikTok content compared with their baseline levels (*p* < 0.001). Importantly, participants exposed both to sexualized and non‐sexualized body‐positive content reported statistically significantly greater body satisfaction compared with their baseline levels (*p* < 0.001 and *p* = 0.044, respectively). After exposure, there were no differences in body satisfaction levels between sexualized and non‐sexualized body positivity conditions (*p* = 0.127). Notably, this suggests that body satisfaction was higher after exposure to both body‐positive conditions (regardless of the presence of sexualization content) when compared to the sexualized beauty ideal condition. There was also a significant condition × time interaction on positive mood (*F*(2, 313) = 12.59, *p* < 0.001, *p* ^*2*^ = 0.07), such that positive mood significantly decreased among those in the sexualized ideal condition (pre vs. post exposure; *p* < 0.001). After content exposure, the sexualized beauty ideal group showed significantly lower positive mood compared with the sexualized body positivity group (*p* = 0.021). Participants' negative mood significantly decreased after exposure to both body positivity conditions (*p*_*s*_ < 0.001). There was also a statistically significant effect of condition on appearance social comparison, (*F*(2, 313) = 15.93, *p* < 0.001, *p* ^*2*^ = 0.09). Participants in the sexualized beauty ideal condition showed greater engagement in social comparison than those in the sexualized body‐positivity group (*p* < 0.001). Overall, body positivity content promoted engagement in downward social comparison (*t*_*s*_ > 7.12, *p*_*s*_ < 0.001). Participants' self‐objectification and intention to get cosmetic surgery did not differ across groups, *F*_*s*_(2, 313) < 0.59, *p*_*s*_ > 0.553
Dhadly et al. (2023) #BoPo: Does viewing body positive TikTok content improve body satisfaction and mood?	*N* = 156 Body positive (*n* = 54) Body‐focused (*n* = 54) Control TikTok videos (*n* = 48) Convenience sampling approach. Participants were recruited from the University's Psychology Participation Pool (SONA system) Of the 229 participants who consented to the study, 25 were excluded for completing the survey too quickly or indicating that they did not see the experimental manipulation. Further, there was limited data from men (*n* = 45) and non‐binary (*n* = 2) participants, so only women were included in analyses Participants reported social media use (anywhere from 2 h to 10+ hours daily), with 30.8% participants using it for 2–4 h/day As part of the cover story, participants were told that researchers were studying how personality and mental health may impact one's enjoyment of TikTok content. Participants were debriefed at the end of the study	Participants were randomized into one of the three conditions. All participants watched TikTok videos for 5 min, which were displayed in the same order for all participants within the same group. Videos for the body positive condition were searched using: “#bodypositive” and “#bodypositivity.” Videos for the body‐focused condition were searched using hashtags: “#bodychecking,” “#bodycheck,” “body check,” “body checking,” and “#outfitoftheweek,” and videos for the control condition were searched using hashtags “#doityourself (#DIY),” “#howto,” and “#nature” Before and after viewing TikTok content, participants completed the Positive and Negative Affect Scale present moment version (PANAS‐20) to assess pre‐ and post‐exposure affect, the Body Image States Scale (BISS‐6) to assess body satisfaction pre‐ and post‐exposure, the Eating Pathology Symptoms Inventory Body Dissatisfaction Subscale (EPSI) to assess body dissatisfaction pre‐exposure	Total sample (M = 18.44, SD = 1.07). Age Range = 18–28	Participants both in the body‐focused condition (*t*(53) = 6.098, *p* < 0.001) and the control condition (*t*(47) = 2.712, *p* = 0.009) showed significantly lower positive affect after being exposed to their corresponding TikTok videos, with pre‐exposure scores being 4.02 higher than post‐exposure scores for the body‐focused condition (95% CI [2.69, 5.34]) and 1.81 higher than post‐exposure scores for the control condition (95% CI [0.47, 3.16]). Conversely, those in the body positive (*t*(53) = 2.301, *p* = 0.025) and control conditions (*t*(47) = 5.38, *p* < 0.001) showed statistically significant lower negative affect after exposure, whereas those in the body focused condition showed significantly higher negative affect after exposure (*t*(53) = 2.156, *p* = 0.036). Finally, those participants in the body positivity condition showed statistically significant greater body satisfaction after exposure (*t*(53) = 5.256, *p* < 0.001), whereas those in the body‐focused condition showed statistically significant lower body satisfaction (*t*(53) = 4.694, *p* < 0.001), and there were no changes identified within the control group after exposure (*t*(47) = 1.503, *p* = 0.140). These findings suggest that the type of content that individuals are exposed to on TikTok can impact positively or negatively their body image, and that even being exposed to control TikTok content can decrease negative affect
Drivas et al. (2024) #WhatIEatInADay: The effects of viewing food diary TikTok videos on young adults' body image and intent to diet	*N* = 316 WIEIAD TikTok videos depicting low‐calorie diets (*n* = —)* WIEIAD TikTok videos depicting high‐calorie diets (*n* = —)* Convenience sampling approach. Participants were recruited from a pool of undergraduate students at a university in the Northeastern United States Of the 437 participants who were recruited for the study, 3 were removed because they failed the manipulation check, 21 were removed because they failed the attention check, and 100 were removed because they did not complete the survey More than half participants (50.9%) reported using TikTok multiple times per day *The original manuscript does not specify how many participants were randomized to each condition.	Participants were randomized to one of the two experimental conditions. Researchers selected five TikTok videos for each condition, but each participant in each condition was only randomly exposed to three out of the five videos to utilize stimulus sampling Participants completed the following questionnaires post‐exposure: a state body appreciation measure adapted from the Body Appreciation Scale (BAS); a revised 5‐item version of the Dieting Intentions Scale (DIS) to assess intent to diet; the three‐item body dissatisfaction subscale measured on Visual Analog Scales (VAS); and an adaptation of the State Appearance Comparison Scale (SACS)	Total sample (M = 19.8, SD = 1.4). Age Range = 18–32	Participants exposed to low‐calorie #WIEIAD videos experienced statistically significant greater upward social comparison (*β* = 0.51, *p* < 0.001, 95% CI [0.59, 0.43]) and body dissatisfaction compared with those exposed to #WIEIAD TikTok videos depicting high‐calorie diets. Further, stronger downward comparison increased positive mood (*β* = 0.20, *p* = 0.002, 95% CI [0.08, 0.33]). Social comparison was a significant mediator between video type (i.e., low‐calorie vs. high‐calorie) and positive mood (*β* = 0.20, *p* = 0.002, 95% CI [0.08, 0.33]). Additionally, positive mood mediated the relationship between social comparison and diet intentions (*β* = 0.07, *p* = 0.003, 95% CI [0.02, 0.13]). Of note, the greater intention to diet found among those who viewed high‐calorie #WIEIAD videos was contrary to what was hypothesized and what previous literature suggests. Finally, there was a significant direct effect of TikTok use on body appreciation (*β* = 0.16, *p* = 0.004, 95% CI [0.27, 0.06])
Fiuza and Rodgers (2023) The effects of brief diet and anti‐diet social media videos on body image and eating concerns among young women	*N* = 421 Diet culture (*n* = 134) Anti‐diet culture (*n* = 130) Neutral (*n* = 134) Panel sampling approach. Women who use social media were recruited via a Qualtrics Survey Panel *n = 23 participants were removed for failing the attention check.	Participants were randomized to one of three conditions and watched two, brief (10–12 s in length), consecutive TikTok videos. The two diet culture videos were selected using the hashtags #whatIeatinaday and #weightlosstips, which depicted young women documenting their eating in a given day (< 1050 cal total). The two anti‐diet videos were selected using hashtags such as #bodypositivity, #bodypositive, and #self‐love, with the message being that women should not avoid certain foods or engage in exercise to look thinner. The two neutral videos were animal videos selected using hashtags such as #funnyanimalvideos Participants completed state measures both pre‐ and post‐exposure. They completed the Visual Analog Scales (VAS) to assess state weight and shape satisfaction, state intuitive eating, state positive mood, and state negative mood; two items to assess state body appreciation; an adaptation of the Intuitive Eating Scale‐2 (IES‐2) to assess state intuitive eating; a single item developed by the research team to assess state restriction and state urges to exercise; the Thin/Low Body Fat Internalization subscale of the Sociocultural Attitudes Toward Appearance Questionnaire (SATAQ‐4R); and the Social Media Rumination Scale (SMRS)	Total sample (M = 19.49, SD = 1.10). Age Range = 18–21	Overall, participants in the diet culture condition showed worse body image and eating‐related outcomes. Those in the anti‐diet condition demonstrated a statistically significant increase in weight and shape satisfaction, *t*(127) = 3.57, *p* < 0.001 and positive mood, *t*(127) = 3.57, *p* < 0.001, whereas those in the diet culture condition showed greater negative mood, although it was not statistically significant (*p* = 0.88). Those in the anti‐diet condition also showed a greater increase in state body appreciation (*p* = 0.015) and state intuitive eating (*p* = 0.005) compared to the body neutral condition. Those in the diet culture condition also showed greater restriction, although not statistically significant (*p* = 0.92), and urges to exercise (*p* = 0.08) compared with the body neutral condition. Finally, social media‐related rumination was a statistically significant trait moderator between video exposure (diet culture vs. the anti‐diet and neutral videos) and state intuitive eating (*B* = 3.17, SE = 1.52, *p* = 0.037), as well as between the association between video exposure and state restriction (*B* = 6.62, SE = 2.88, *p* = 0.02). Conversely, thin ideal internalization was not a statistically significant moderator in the association between video exposure and state intuitive eating (*p* = 0.30 and *p* = 0.58) or state restriction (*p* = 0.60 and *p* = 0.58)
Galway and Gammage (2025) The effect of viewing #whatieatinaday TikTok videos with and without calories on body‐related shame, guilt, envy, and intentions to diet in young women	*N* = 335 #WIEIAD videos with calories (*n* = 110) #WIEIAD videos without calories (*n* = 113) Travel (control) videos (*n* = 112) Panel sampling approach. Participants were recruited through Cloud Research Connect, an online crowd sourcing tool Of the 351 participants who completed the survey, three participants were removed from the study because they failed attention checks, one participant was removed due to inconsistent answers, seven were removed because they did not meet inclusion criteria, and 5 were removed because they completed the survey too fast (less than 14 min) Across conditions, most participants reported using TikTok for 1 h daily The cover story went as follows: “Participants were recruited for a study titled ‘social media use, self‐perceptions and mood.’” At the end of the study, participants were informed about the use of deception	Participants were randomly assigned to one of the three experimental conditions. For the #WIEIAD videos without calories condition, videos were selected using hashtags “#whatieatinaday” and “#WIEIAD,” as well as “what I eat in a day” on the TikTok search bar. These videos did not display calorie counts but showed consumption of an estimated daily intake greater than 1200 cal. For the #WIEIAD videos with calories condition, the same videos as the “no calorie” condition were used, but researchers estimated the calories for each item consumed using an online calorie counter. Calories were included in each video. Finally, for the control condition, travel videos were selected using “#travel” in TikTok, as well as travel on the TikTok search bar. Videos did not include food or appearance‐related content Participants completed the Visual Analogue Scales (VAS) to assess changes in state body image; the Shame and Guilt subscales of the Body and Appearance Related Self‐conscious Emotions scale (BASES) to assess body‐related shame and guilt; the Appearance subscale of the Body Envy scale to assess state appearance‐related envy, two items to assess state upward appearance comparisons; the Intentions to Change Diet subscale from the Diet and Exercise Intentions questionnaire; the Trait Self‐Compassion Scale—short form (SCS‐SF) to assess how participants cope with internalized flaws; the International Physical Activity Questionnaire—Short Form (IPAQ‐SF) to assess physical activity; and the Internalization of the Thin/Low Body Fat Ideal subscale from the Sociocultural Attitudes Toward Appearance Questionnaire‐4R (SATAQ‐4R)	Total sample (M = 25, SD = 3.24). Age Range = 18–30	There were no statistically significant differences across #WIEIAD conditions (i.e., the one that included calories and the one that did not) in body‐related envy (*p* = 0.583, 95% CI [−6.51, 3.72]) or intention to change their diet (*p* = 0.751, 95% CI [−0.43, 0.32]). However, participants who were exposed to the calorie videos reported higher levels of body‐related envy (*p* = 0.007, 95% CI [2.10, 11.45]) and intention to change their diet (*p* = 0.001, 95% CI [−0.99, −0.31]), respectively, compared with those who viewed travel (control) TikTok videos. Of note, appearance comparisons mediated the relationship between video condition (#WIEIAD conditions vs. travel control videos) and all the tested outcomes including body‐related shame, *b* = 2.89, *t* = 5.49, *p* < 0.001, 95% CI [1.85, 3.92], body‐related guilt, *b* = 2.26, *t* = 4.06, *p* = < 0.001, 95% CI [1.16, 3.35], body‐related envy, *b* = 3.99, *t* = 7.34, *p* < 0.001, 95% CI [2.92, 5.06], and intentions to change diet, *b* = 0.29, *t* = 7.32, *p* < 0.001, 95% CI [0.21, 0.38], but self‐compassion was not a statistically significant moderator for any of these relationships. Interestingly, there were no differences in any of the outcomes between participants who were exposed to #WIEIAD videos with calories vs. those who were exposed to #WIEIAD videos without calories
Gurtala and Fardouly (2023) Does medium matter? Investigating the impact of viewing ideal image or short‐form video content on young women's body image, mood, and self‐objectification	*N* = 211 Appearance‐ideal Instagram images (*n* = 52) Appearance‐ideal TikTok videos (*n* = 54) Appearance‐ neutral Instagram images (*n* = 52) Appearance‐neutral TikTok videos (*n* = 53) Convenience sampling approach. Participants were recruited from a university participant pool The cover story presented the study as an “online study evaluating posts on social media” Of the 215 participants who completed the study, four were removed from analyses because they failed the attention checks Most participants reported spending 2–3 h on social media daily, and specifically, 1–2 h on TikTok daily	Participants were randomized to one of the four conditions. Those exposed to the appearance‐ideal (experimental) conditions watched contemporary societal beauty ideals content, including attractive faces (i.e., large eyes, full lips, and small nose), thin, and toned bodies—typically depicted in bikinis, fitness clothing, or form‐fitting clothing. Participants exposed to the appearance‐neutral (control) conditions watched images and videos obtained from public interior design accounts. All stimuli across conditions were randomly presented for 10 s each All participants completed the state appearance satisfaction and negative mood scale both pre‐ and post‐exposure, a modified version of the Twenty Statements Test to assess state self‐objectification both pre‐ and post‐exposure, the Internalization‐Thin/Low Body Fat, Internalization‐Muscular, and Internalization‐General Attractiveness trait subscales of the Sociocultural Attitudes Toward Appearance Questionnaire‐4‐Revised (SATAQ‐4R) post‐exposure. Further, only participants in the experimental conditions (post‐exposure) completed the State Appearance Comparison Scale (SACS) to assess the frequency and duration of appearance comparison, items of the perceived attainability measure to assess perceived attainability of the appearance ideals, and a scale developed by the researchers to assess perceived appearance enhancement	Total sample (M = 19.190, SD = 1.803). Age Range = 17–28	Participants exposed to both appearance‐ideal videos and images showed increased appearance dissatisfaction, *F*(1, 208) = 39.30, *p* < 0.001, negative mood, *F*(1, 205) = 7.06, *p* = 0.009, self‐objectification, *F*(1, 187) = 19.58, *p* < 0.001, and internalization of appearance ideals, *F*(1, 205) = 62.062, *p* < 0.001, *η* ^2^_*p*_ = 0.232, compared with those who viewed appearance‐neutral videos and images. Across the appearance ideal experimental conditions, there were no statistically significant differences in participants' appearance comparison frequency, *F*(1, 104) = 0.002, *p* = 0.964, *η* ^2^_*p*_ < 0.001 or direction, *F*(1, 104) = 0.002, *p* = 0.968, *η* ^2^_*p*_ < 0.001, or in their perceived attainability of the target women's appearances, *F*(1, 104) = 0.506, *p* = 0.478, *η* ^2^_*p*_ = 0.005. The only difference found between the experimental conditions was that participants described the images to be more edited/enhanced than the videos, *F*(1, 102) = 10.814, *p* = 0.001, *η* ^2^_*p*_ = 0.096. Accordingly, TikTok videos were perceived as less edited, which elicited lower appearance satisfaction among participants. A potential explanation is that participants could have compared their bodies with more “real” individuals when watching the videos vs. seeing the images
Joiner et al. (2023) The effect of different types of TikTok dance challenge videos on young women's body satisfaction **(Study 1)**	*N* = 262 Thin dancers (*n* = —)* Large dancers (*n* = —)* Amusing animal videos (*n* = —)* Convenience sampling approach. Participants were recruited via social media and Prolific, a crowd sourcing recruitment platform Thirteen participants were removed from the analyses because they completed the survey faster than the set threshold (i.e., 240 s) Most participants reported spending 1–2 h on TikTok daily *The original manuscript does not specify how many participants were randomized to each condition.	Participants were randomized into one of the three conditions. No specific cover story is mentioned, but researchers did not use deception with participants. In each condition, participants were exposed to 6 TikTok videos for approximately 130 s in total. In the two experimental conditions, all depicted women in the videos were Caucasian, and they were wearing bikinis, shorts, leggings, tracksuit bottoms, crop tops and t‐shirts. Videos in the two experimental conditions were matched in terms of type of clothing Participants completed Visual Analogue Scales (VAS) to assess body satisfaction both pre‐ and post‐exposure through three items: weight satisfaction, overall appearance satisfaction, and body shape satisfaction. After participants were exposed to the content, they were asked to rate the attractiveness of the dancer or how amusing the animals in the control videos were	Total sample (M = 20.09, SD = 2.45). Age Range = 18–25	Female participants exposed to videos of large dancers showed a statistically significant increase in body satisfaction, *t*(88) = 2.485, *p* = 0.015, *d* = 0.26, a well as those exposed to the control condition, *t*(83) = 4.424, *p* < 0.001, *d* = 0.48 Participants' body dissatisfaction levels decreased after watching thin dancers, but this difference was not statistically significant, *t*(88) = 1.544, *p* = 0.126, *d* = 0.16. Additionally, participants rated thin dancers (M = 7.81, SD = 3.40) as statistically significantly more attractive than large dancers (M = 5.73, SD = 1.96), *t*(1, 171) = 4.89, *p* < 0.001, *d* = 0.74. Further, watching amusing animal videos (control condition) also had a positive impact on body satisfaction, but those exposed to large dancer videos showed a greater increase in body satisfaction than those in the control condition
Joiner et al. (2023) The effect of different types of TikTok dance challenge videos on young women's body satisfaction **(Study 2)**	*N* = 280 Thin dancers (*n* = —)* Large dancers (*n* = —)* Animal videos + oversized animal costumes (*n* = —)* Convenience sampling approach. Participants were recruited via Prolific, a crowd sourcing recruitment platform Fourteen participants were removed from the analyses because they completed the survey faster than the set threshold (i.e., 240 s) Most participants reported spending 1–2 h on TikTok daily *The original manuscript does not specify how many participants were randomized to each condition.	Participants were randomized into one of the three conditions. No specific cover story is mentioned, but researchers did not use deception with participants. Each participant viewed 6 videos (for approximately 100 s) regardless of the condition they were assigned to. Although the same criteria were used to select the videos, those in study 2 were different from those of study 1. Researchers ensured that all videos in this study had the same music in the background. Those assigned to the control condition were exposed to both animal videos and videos of people dressed in oversized animal costumes Participants completed Visual analogue scales (VAS) to assess body satisfaction both pre‐ and post‐exposure through three items: weight satisfaction, overall appearance satisfaction, body shape satisfaction, as well as a mood measure. After participants were exposed to the experimental content, they were asked to rate the dancer's attractiveness and thinness through two Likert‐scale type questions developed by the researchers. Those exposed to the control content were asked how enjoyable the video was and how enjoyable the music was. After exposure, only those in the experimental conditions completed the 11 item Physical Appearance Comparison Scale—Revised (PACS‐R) to assess state appearance comparison; they assessed the direction of social comparison through a single question. Participants also completed the thin/low body fat subscale of the Sociocultural Attitudes Toward Appearance Questionnaire–Version 4 (SATAQ‐4) to assess thin ideal internalization, the body satisfaction scale used in study 1, and the State Appearance Comparison scale (SACS)	Total sample (M = 21.29, SD = 1.80). Age Range = 18–25	The findings were consistent with Study 1's results for the most part. Specifically, body satisfaction significantly increased post‐exposure in the large dancers' condition, *t*(94) = 2.501, *p* = 0.014, *d* = 0.26. Body satisfaction significantly decreased after watching thin dancers in this study, *t*(92) = 2.39, *p* = 0.019, *d* = 0.24. Of note, internalization of the thin ideal (*B* = 1.12, SE = 0.78, *p* = 0.154) or trait appearance comparison (*B* = 0.82, SE = 0.71, *p* = 0.246) did not moderate these findings. There was a statistically significant difference between the two experimental conditions regarding the direction of social comparison, *F*(1, 186) = 134, *p* < 0.001, *η* ^2^_*p*_ = 0.41 indicating that participants who watched thin dancer videos engaged in upward social comparison to a greater extent, whereas those who watched large dancer videos engaged in downward social comparison. Social comparison mediated the effect of video type on body satisfaction, *F*(1, 186) = 134.35, *p* < 0.001, *η* ^2^_*p*_ = 0.419. Finally, there were no significant changes in body satisfaction within the control condition, *t*(88) = 1.152, *p* = 0.252, *d* = 0.12 (which is inconsistent with study 1). Participants also rated thin dancers (M = 6.20, SD = 1.17) as statistically significantly more attractive than large dancers (M = 5.53, SD = 1.08), *t*(186) = 4.09, *p* < 0.001, *d* = 0.62, as well as statistically significantly thinner (M = 6.38, SD = 0.70) than large dancers (M = 3.62, SD = 0.63), *t*(186) = 28.44, *p* < 0.001, *d* = 4.14
Joiner et al. (2023) The effect of different types of TikTok dance challenge videos on young women's body satisfaction **(Study 3)**	*N* = 375 Thin dancers (*n* = —)* Large dancers (*n* = —)* Convenience sampling approach. Participants were recruited via Prolific, a crowd sourcing recruitment platform Thirteen participants were removed from the analyses because they completed the survey faster than the set threshold (i.e., 200 s) This study (as opposed to the two previous ones) concealed the true purpose of the study and only administered post‐test measures. Participants were told that this study intended to investigate “how engaging are TikTok videos.” At the end of the study, they were told the real purpose of the study Most participants reported spending 1–2 h on TikTok daily *The original manuscript does not specify how many participants were randomized to each condition.	Participants were randomized to one of the two conditions. Participants in each condition were exposed to six TikTok videos. These videos were different from those from studies 1 and 2. Across both conditions, videos were matched so that women's type of clothing was similar. Participants across conditions were exposed to videos for approximately 90 s Participants were then asked how engaging each video was (in a Likert‐type scale question developed by researchers), and they also completed the same body satisfaction scale from the two previous studies	Total sample (M = 22.54, SD = 1.84). Age Range = 18–25	This study confirmed that participants' body satisfaction increased after watching large dancers (M = 54.47, SD = 25.57) compared with those who watched thin dancers (M = 47.42, SD = 26.45), *t*(373) = 2.62, *p* = 0.009, *d* = 0.27
Kilby and Mickelson (2025) Combating weight‐stigmatization in online spaces: the impacts of body neutral, body positive, and weight‐stigmatizing TikTok content on body image and mood	*N* = 326 Body positivity (*n* = 81) Body neutrality (*n* = 82) Thin ideal/weight stigmatizing (*n* = 79) Travel (control) videos (*n* = 84) Convenience sampling approach. Participants were recruited via Connect Cloud Research, a professional participant platform. Participants were debriefed at the end of the study and received a resource page with mental health and ED support Most participants reported daily use of TikTok to be between 30 min—2 h	Participants were randomized into one of the four conditions. Across conditions, participants were exposed to a 5‐min video (which included approximately 20 videos, with an average length of 20 s each). Researchers looked for specific hashtags to identify the body acceptance TikTok videos (e.g., body neutral: #bodyneutrality, #bodyneutral; body positive: #bodypositivity, #bodypositive). Videos that featured content that overlapped across these two conditions were not included. The content across these two conditions matched: both explained body neutrality or body positivity, mindfulness practices, songs, and the purpose of food, physical exercise, and/or clothes. Weight‐stigmatization videos also matched the content presented in the body acceptance conditions, but this condition was exposed to food for dieting purposes, physical exercise for weight loss, clothing for “flattering” or “slimming” purposes, and mantras encouraging thin idealization or promoting weight stigma. Explicit ED content was excluded from all conditions. Participants in the control condition were exposed to travel destinations and scenic shots (without content creators or any other people in the videos) Pre‐exposure, participants were asked to complete the Stunkard Figure Rating Scale to assess participants' perception of their body silhouette; the Positive and Negative Affect Scale (PANAS) to assess mood; the 7‐item Self‐Objectification Beliefs and Behaviors Representing Self Subscale to assess self‐objectification; and the 7‐item Functionality Appreciation Scale (FAS) to assess functional appreciation. Post‐exposure, participants were asked to complete three Likert‐type questions developed by researchers to assess the existence of state appearance thoughts and their frequency experienced during exposure, as well as the likelihood of continuing to watch body neutrality and body positivity content outside of the study	Total sample (M = 35.01, SD = 14.96). Age Range = 18–67	Exposure to both body neutrality and body positivity content had promising effects on participants' body image and mood. Body dissatisfaction was statistically significantly lower, *F*(3, 320) = 8.14, *p* < 0.001, *η* ^2^_*p*_ = 0.071 in the body positive (MD = 10.86, SD = 2.38, *p* < 0.001), body neutral (MD = 9.41, SD = 2.38, *p* = 0.001), and control (MD = 7.14, SD = 2.36, *p* = 0.016) conditions compared with the weight stigma condition after exposure. Further, participants in the body positive condition showed statistically significant greater functional appreciation (MD = 0.16, SD = 0.05, *p* < 0.001), as well as lower self‐objectification (MD = −0.17, SD = 0.07, *p* = 0.010), body dissatisfaction, and negative affect (MD = −0.20, SD = 0.06, *p* < 0.001) (pre vs. immediate post exposure). Similarly, those exposed to the body neutrality videos showed statistically significant higher functional appreciation (MD = 0.14, SD = 0.05, *p* = 0.003), as well as lower self‐objectification (MD = −0.22, SD = 0.07, *p* = 0.001) and negative affect (MD = −0.20, SD = 0.06, *p* < 0.001; pre‐ vs. immediate post‐exposure). There was a marginal increase in positive affect after exposure to body positivity content (MD = 0.13, SD = 0.07, *p* = 0.051). In addition, participants in both the body positive (*p* < 0.001) and body neutral groups (*p* < 0.001) critically reported more positive thoughts compared with the other conditions. Finally, results showed a marginal three‐way interaction, *F*(3, 312) = 2.52, *p* = 0.058, *η* ^2^_*p*_ = 0.02—indicating that the effects of each condition on body dissatisfaction differed among gender identity, whereas the effects on positive affect varied by participants' body silhouettes (i.e., smaller, mid‐sized, and larger). Further research is warranted to better understand the differential positive impacts of body neutrality and body positivity content on individuals' body image and mood
Li et al. (2025) They are more beautiful than me! How social media use increases women's body‐related envy and cosmetic surgery consideration **(Study 1b)**	*N* = 110 TikTok use priming condition/appearance‐relevant content (*n* = 55) Control (not absolutely neutral) condition (*n* = 55) Convenience sampling approach. Participants were recruited from a university through WeChat Moments or WeChat groups Participants were told that this was a study about “women's appearance self‐perception,” and that they needed to carefully browse the content about yoga pants within 5 min because it would be tested in the subsequent task. They were asked to pay careful attention to the text and image. Then, participants were debriefed	Participants were equally split into each of the two conditions (no randomization is mentioned). Participants in the experimental/priming condition were exposed to women's physical appearance‐relevant content, specifically, 10 pictures of women wearing yoga pants and an introductory text. Participants in the control condition were presented with a PDF document with 10 images of yoga pants alone and the same introductory text After exposure, participants were asked to report the extent to which they felt resentful, longing for, jealous, and covetous on a 5‐point scale (modified version of the Dispositional Envy Scale; DES)	Total sample (M = 21.26, SD = 0.95). Age Range = 19.33–23.17	Participants in the TikTok use priming condition (M = 6.44, SD = 0.66) indicated that the priming condition presented statistically significantly greater appearance‐related content compared with the control condition content (M = 3.65, SD = 1.81), *t*(108) = 10.72, *p* < 0.001, *d* = 2.04. Further, post‐exposure, participants in the TikTok use priming condition (M = 16.82, SD = 2.07) showed statistically significantly higher female body‐related envy (i.e., resentful, longing for, jealous, and covetous) than the control condition (M = 12.96, SD = 3.96), *t*(108) = 6.40, *p* < 0.001, *d* = 0.81
Li et al. (2025) They are more beautiful than me! How social media use increases women's body‐related envy and cosmetic surgery consideration **(Study 3)**	*N* = 200 TikTok use priming condition/appearance‐relevant content (*n* = 100) Control (not absolutely neutral) condition (*n* = 100) Convenience sampling approach. Participants were recruited from the Credamo platform At the end of the study, participants were debriefed (similar cover story and debrief process as in study 1b)	Participants were equally split into each of the two conditions (no randomization is mentioned). The samples in studies 1b and 3 were independent from each other, so participants in study 3 were exposed to the same stimuli as study 1b. In this study, researchers bolded the font to remind participants that they should not only pay attention to the text but also to the pictures Participants completed the same body‐related envy measure as in study 1b. Participants also completed the five‐item consideration subscale of the Acceptance of Cosmetic Surgery Scale (ACSS) to examine the extent to which they would consider cosmetic surgery to alter their physical appearance. They also completed the Chinese version of the Rosenberg Self‐Esteem Scale (RSES), as well as the Envy Scale to measure participants' dispositional envy	Total sample (M = 32.03, SD = 7.86). Age Range = 18.08–57.00	Participants exposed to the TikTok use priming condition (M = 6.24, SD = 0.92) reported the presence of statistically significantly higher appearance‐relevant content than those in the control condition (M = 3.21, SD = 1.86), *t*(198) = 14.59, *p* < 0.001, *d* = 2.06. Participants exposed to the priming TikTok condition reported statistically significantly higher female body‐related envy (M = 16.11, SD = 2.25) than those in the control condition (M = 12.39, SD = 4.18), *t*(198) = 7.85, *p* < 0.001, *d* = 1.11, as well as statistically significantly higher cosmetic surgery consideration (M = 4.19, SD = 0.50) compared with those in the control condition (M = 2.75, SD = 1.20), *t*(198) = 11.12, *p* < 0.001, *d* = 1.57. Finally, there was a statistically significant mediation of body‐related envy between the experimental condition, and women's cosmetic surgery consideration, *F*(7, 192) = 30.21, *R* ^2^ = 0.52, *p* < 0.001
Limniou et al. (2025) TikTok fitspiration and fitness ideal internalization: Gender differences in self‐esteem and body satisfaction	*N* = 274 All participants were exposed to neutral content and Fitspiration content (within‐subjects) Opportunity sampling approach. Participants were recruited through social media and an internal recruitment scheme Participants' average daily TikTok use was greater than 52 min Participants were debriefed at the end of the study and received resources and researchers' contact information in case needed	Participants were first exposed to neutral TikTok videos (i.e., 47 s of three travel‐themed videos searched by researchers under “#travel” and included relaxing videos). Then, they were exposed to Fitspiration content, which included six fitness‐focused videos (three TikTok videos per gender). Researchers identified these videos under “#fitspiration” or “#fitspo,” illustrating individuals in fitness clothing completing exercises in a gym while posing for the camera, emphasizing the fit parts of their bodies. Male participants were exposed to these videos for 54 s, whereas females were exposed to these videos for 48 s Participants were asked to complete the Ideal Internalization Scale to assess the extent to which they felt pressured by TikTok to comply with fit ideals, the Physical Appearance Scale (PACS‐R) to assess participants' tendency to compare their physical appearance to that of others, and the State Self‐Esteem Scale (SSES) to assess self‐esteem twice (after exposure to each set of videos). Body satisfaction was also assessed twice (after the exposures) through a single item of the Body Satisfaction Scale (BSS)	Total sample (M = 21.8, SD = 7.64). Age Range = 18–62	Body satisfaction levels significantly decreased after brief exposure to fitspiration content (M = 50.0, SD = ±21.94), *t*(273) = 7.344, *p* < 0.001, Cohen's *d* = 0.44, 95% CI [0.32, 0.56], and women had lower body satisfaction levels than men before, *F*(1, 271) = 4.702, *p* = 0.031, partial *η* ^2^ = 0.017, 95% CI [0.001, 0.055], and after, *F*(1, 271) = 6.804, *p* = 0.010, partial *η* ^2^ = 0.024, 95% CI [0.004, 0.067] exposure. Gender significantly affected results—there was a significant gender × condition interaction, with gender being a significant predictor of post‐exposure body satisfaction (*β* = −3.02, *p* = 0.012, 95% CI [−5.38, −0.66]) and state self‐esteem (*β* = −4.42, *p* < 0.001, 95% CI [−6.52, −2.32]). Lower scores of self‐esteem were reported among women compared with men both pre‐*F*(1, 271) = 14.608, *p* < 0.001, partial *η* ^2^ = 0.051, 95% CI [0.020, 0.096] and post‐*F*(1, 271) = 15.887, *p* < 0.001, partial *η* ^2^ 0.055, 95% CI [0.022, 0.100] exposure to fitspiration. However, men also showed a decrease in body satisfaction after exposure (M = 56.8, SD = 18.37; M = 54.3, SD = 19.48). There were no statistically significant differences between TikTok usage (e.g., number of accounts followed) and physical appearance comparison (Adjusted *R* ^2^ = 0.029, *F*(5, 196) = 1.601, *p* = 0.160). Finally, active engagement with TikTok led to greater fit internalization as opposed to passive use
Pryde and Prichard (2022) TikTok on the clock but the #fitspo don't stop: The impact of TikTok fitspiration videos on women's body image concerns	*N* = 120 Fitspiration TikTok videos (*n* = 61) Art TikTok (control) videos (*n* = 59) Convenience sampling approach Participants were recruited from Flinders University first‐year psychology pool and from online sources (e.g., Facebook groups, Facebook advertising, and the university's volunteer and research webpage)	Participants were randomized to one of the two conditions All videos were sourced from public profiles on TikTok. The fitspiration videos were found using #fitspo, and the art videos were found using #art. A pilot study rated if the videos were representative of the condition, their video and audio quality, and the extent to which each video contained a focus on overall appearance or body parts. Each final set contains 10 min of content	Total sample (M = 21.06, SD = 2.40). Age Range = 18–25	Viewing fitspiration TikTok videos led to statistically significant greater negative mood (*p* = 0.041, 95% CI of the difference = −6.71 to −0.15) compared with viewing art TikTok videos (*p* = 0.087, 95% CI of the difference = −0.43 to 6.24). Viewing fitspiration videos also led to greater appearance comparison (M = 4.61, SD = 1.73) compared with viewing art videos (M = 2.38, SD = 1.46), *t*(118) = −7.61, *p* < 0.001, *d* = 1.39 In addition, viewing fitspiration TikTok videos indirectly increased body dissatisfaction compared with viewing art TikTok videos, *b* = 6.48, 95% CI [2.75, 10.20], *p* < 0.001. Findings regarding body dissatisfaction and negative mood levels were not moderated by trait fit ideal internalization but were mediated by state appearance comparison, *b* = 4.26, 95% CI = [1.01, 7.85] (i.e., viewing fitspiration TikTok videos increased women's body dissatisfaction via appearance comparisons). There was a non‐significant increase in body dissatisfaction when comparing baseline with post‐exposure scores in the fitspiration condition (*p* = 0.123, 95% CI of the difference = −4.65 to 0.56), whereas there was a significant decrease in body dissatisfaction within the art control group, (*p* < 0.001, 95% CI of the difference = 1.96–7.25)
Seekis and Lawrence (2024) The effect of TikTok body neutrality content on young women's self‐compassion	*N* = 189 Body neutrality (*n* = 63) Thin ideal (*n* = 63) Art control (*n* = 63) Convenience sampling approach. Participants were students recruited for course credit Participants were told that the purpose of the study was to investigate the effects of different types of TikTok content on memory recall and emotional processing Of *N* = 190, 1 participant was removed from analyses due to incorrectly answering two out of three attention checks	Participants were randomized to one of the three conditions Video content was searched and selected from hashtags associated with body neutrality (e.g., #bodyneutrality), thin ideal (e.g., #workouttiktok), and art (e.g., #artpainting). Two pilot studies assessed how representative the TikToks were of the condition, their overall quality, and timing of the videos. Each condition featured a 12‐min compilation video of TikToks	Total sample (M = 19.25, SD = 1.98). Age Range = 17–28	Self‐compassion was higher among participants who were exposed to TikTok body neutrality content (M = 3.39, SD = 0.63; i.e., videos depicting diverse body shapes, including diverse women content creators discussing aspects of body neutrality, clothes, and healthy exercise) compared with those who viewed thin ideal content (M = 2.73, SD = 0.87, *t*(124) = 6.01, *p* < 0.001, *d* = 0.87; i.e., videos depicting lean and toned content creators getting ready for events and doing exercise to control their weight/shape) and those who were exposed to art control content (M = 3.05, SD = 0.61, *t*(124) = 2.04, *p* = 0.042, *d* = 0.55; i.e., videos depicting painting techniques). Importantly, participants did not differ on pretest levels of self‐compassion across groups, *F*(2, 186) = 1.58, *p* = 0.208, *η* ^2^_*p*_ = 0.017
Seekis and Lawrence (2023) How exposure to body neutrality content on TikTok affects young women's body image and mood	*N* = 189 Body neutrality (*n* = 63) Thin ideal (*n* = 63) Art control (*n* = 63) Convenience sampling approach. Participants were recruited at a large Australian public university Researchers utilized a cover story to disguise the true purpose of the study, but no specific details were provided Of *N* = 190, 1 participant was removed from analyses due to incorrectly answering two out of three attention checks Participants' daily use of social media averaged 3.47 h (SD = 1.31)	Participants were randomized to one of the three conditions Video content was searched and selected from hashtags associated with body neutrality (e.g., #bodyneutrality), thin ideal (e.g., #workouttiktok), and art (e.g., #artpainting). Two pilot studies assessed how representative the TikToks were of the condition, their overall quality, and timing of the videos. Each condition featured a 12‐min compilation video of TikToks	Total sample (M = 19.25, SD = 1.98). Age Range = 17–28	At post‐test, the body neutrality condition showed higher levels of body functionality appreciation, *F*(2, 185) = 59.09, *p* < 0.001, *η* ^2^_*p*_ = 0.39, than the thin ideal, *t*(124) = 10.83, *p* < 0.001, *d* = 1.02, and art control groups, *t*(124) = 4.60, *p* < 0.001, *d* = 0.39. The neutrality condition also showed higher levels of body satisfaction, (*F*(2, 185) = 37.18, *p* < 0.001, *η* ^2^_*p*_ = 0.29, compared to the thin ideal, *t*(124) = 8.61, *p* < 0.001, *d* = 0.76, and art control groups, *t*(124) = 3.97, *p* < 0.001, *d* = 0.38). Even though the body neutrality condition showed a higher positive mood than the thin ideal group, *t*(124) = 6.79, *p* < 0.001, *d* = 0.71, there were no significant differences between the body neutrality and art control groups, *t*(124) = 2.07, *p* = 0.04, *d* = 0.29. Participants in the thin ideal condition showed greater upward appearance comparison than those in the body neutrality, *t*(124) = 7.85, *p* < 0.001, *d* = 1.23, and art control conditions, *t*(124) = 13.02, *p* < 0.001, *d* = 2.49. Finally, women in the body neutrality group reported more positive thoughts about their appearance while watching the videos compared with the thin ideal condition, *t*(116) = 6.38, *p* < 0.001, *d* = 1.17. Of note, there were no pre‐test differences in body functionality appreciation (*F*(2, 186) = 0.40, *p* = 0.673), body satisfaction (*F*(2, 186) = 0.02, *p* = 0.979), or positive mood across groups (*F*(2, 186) = 0.11, *p* = 0.900)
Strickland et al. (2025) TikTok and disordered eating: Delineating temporal associations and effects of a ban	*N* = 252 Pre‐ban group (*n* = 84) Post‐ban group (*n* = 168) Convenience sampling approach. Participants were recruited from a large public university Of *N* = 255 enrolled participants, 3 were excluded due to missing data or age over 24 After completing follow‐up surveys, participants were debriefed. They received information about the study background, design, and hypotheses	After data collection began, an institutional ban was introduced, introducing a quasi‐experimental design. Therefore, there was a group of participants who completed baseline surveys before vs. after the ban was instituted Participants completed the following measures at baseline and follow‐up: the Eating Attitudes Test (EAT‐26) to assess disordered eating attitudes and behaviors and a TikTok questionnaire created by the researchers to measure #WIEIAD engagement	Total sample (M = 19.60, SD = 1.26). Age Range = 18–24	Disordered eating attitudes and behaviors scores at baseline were significantly positively correlated with those at a 9‐week follow‐up (*r*(252) = 0.80, *p* < 0.001), as well as with #WIEIAD engagement at baseline (*r*(252) = 0.55, *p* < 0.001) and follow‐up (*r*(252) = 0.49, *p* < 0.001). Further, both #WIEIAD engagement and disordered eating attitudes and behaviors scores increased significantly from baseline to follow‐up (*B*(SE) = 3.04 (0.76); *β* = 0.23; *t* = 3.99, *p* < 0.001). However, (and contrary to prior literature), #WIEIAD engagement did not predict disordered eating (*B*(SE) = 0.003 (0.042); *β* = 0.04; *t* = 0.97, *p* = 0.33). A university ban that aimed to decrease engagement with/time spent in TikTok did not reduce engagement with #WIEIAD videos (*p* = 0.73). Further, disordered eating attitudes and behaviors scores were significantly higher over time after the university ban was effective (*p* = 0.007). These findings support the promising landscape for interventions developed within social media platforms (i.e., TikTok) as opposed to attempting to limit individuals' use/engagement with TikTok

*Note:* Table with details and main findings from articles included in the review. For other statistical symbols reported, please refer to the original paper.

Abbreviations: ED, eating disorder; M, mean; *N*, sample size; *n*, sub‐sample size; *p*, *p* value; SD, standard deviation.

### 3.1 Content Analyses

Nineteen articles reported content or thematic analyses of TikTok videos and comments. Three papers examined nutrition, dieting, and eating‐related videos (Minadeo and Pope 2022; Munro et al. 2024; Raiter et al. 2023). These videos often portray body checking, promotion of the thin ideal (Munro et al. 2024), and weight loss glorification (Minadeo and Pope 2022), with contradictory (both positive and negative) messages about body image and diet culture (Raiter et al. 2023). Most of the videos analyzed in these articles depicted White, female adolescents and young adults and presented a weight‐normative view of health, with less than 3% of videos coded as weight‐inclusive in one study (Minadeo and Pope 2022). In two papers analyzing fitness and “fitspiration” videos, messages of objectification, commercialism, and inner positivity were common (Nuhn et al. 2025; Pryde et al. 2024). Three papers examined TikTok eating trends (e.g., #WhatIEatInADay) and found an overwhelming portrayal of the thin ideal (Hu et al. 2023; Davis et al. 2023; Wu et al. 2025). Finally, Rogers et al. (2025) found that nearly half of the videos with the hashtag #PostpartumBody involved negative body image themes, and roughly, 25% of videos featured both positive and negative body image themes.

Four studies examined body positivity and/or neutrality. The most popular theme in body positivity videos was body acceptance and love, and the most popular body neutrality theme was size inclusivity (Aubrey et al. 2024). A theme present in #bodyneutral videos was that body neutrality is superior to body positivity (Mancin et al. 2024). Further, body positivity content contains sexually objectifying (Harriger et al. 2023) and eating disordered content (Hallward et al. 2023).

Five studies examined ED recovery content, which ranged from pro‐recovery encouragement and diet culture criticisms to pro‐ED content (Greene and Norling 2023; Greene et al. 2023; Herrick et al. 2021; Kells et al. 2025; Lookingbill et al. 2023). Greene et al. (2023) found differences between ED recovery hashtags; for example, diet culture was a key aspect of many #bedrecovery posts, whereas #orthorexiarecovery posts tended to focus on critiques of diet culture. Regarding user engagement, #anarecovery posts received the most likes (Greene et al. 2023); the number of shares was significantly lower in videos that included diet culture critique than those that did not; and views and shares were higher in posts with body monitoring than in those without (Greene and Norling 2023), suggesting that some videos may reinforce maladaptive ED behaviors. Common themes across the ED recovery community TikTok videos included health and support, ED experiences, transformations, and educational content (Herrick et al. 2021). Finally, a reflexive thematic analysis of comment sections from TikToks examining mental illness‐related discourse, including EDs, revealed themes of self‐diagnosis and emotional contagion, suggesting that the algorithm might lead users to specific content related to them (Romann and Oeldorf‐Hirsch 2025).

### 3.2 Observational Studies

Eighteen studies, reported in 17 articles, examined the relationship between TikTok and body image with observational designs. Fourteen papers found an association between TikTok use and negative body image (Ariana et al. 2024; Auf et al. 2023; Caravelli et al. 2025; Dahlgren et al. 2024; Dajches et al. 2024; Dondzilo et al. 2024; Li et al. 2025; Malhotra et al. 2025; Mink and Szymanski 2022; O'Connor et al. 2024; Pan et al. 2023; Primo‐Simões et al. 2025; Sagrera et al. 2022; Salomon et al. 2025). Compared to social media users who prefer Facebook and Instagram, those who prefer TikTok had higher levels of dysmorphic concern (Primo‐Simões et al. 2025). Mink and Szymanski (2022) found that TikTok use was indirectly related to body dissatisfaction through upward appearance comparison. Interestingly, among medical students, TikTok use was found to be associated with greater body‐esteem (Pop et al. 2022). Similarly, in a longitudinal examination, TikTok use did not predict internalized beauty ideals or body image self‐discrepancy (Maes and Vandenbosch 2022).

The literature also highlights discrepancies regarding passive and active TikTok use. Passive use (i.e., viewing only) predicted body‐related envy, while active use (i.e., posting) did not (Li et al. 2025). Passive use was negatively associated with female users' body‐esteem, but active use was positively associated with body‐esteem (Pan et al. 2023). However, specific aspects of TikTok use that are “active,” like following celebrities and using facial filters, were significantly and positively associated with body dissatisfaction and body image concerns, respectively (Caravelli et al. 2025; Dajches et al. 2024). Additionally, those active on TikTok were more likely to seek content related to body positivity/neutrality (Sanzari et al. 2023).

Seven studies explored the relationship between TikTok use and ED behaviors. Only one study found no association between TikTok use and disordered eating among adolescents (López‐Gil et al. 2024). Usta Ulutaş and Okan Bakır (2025) found that TikTok use was significantly related to increased orthorexia nervosa tendency. Griffiths et al. (2024) examined the FYP and TikTok data (e.g., “likes” and hashtags) from TikTok users with EDs and healthy controls and found that participants with EDs viewed more appearance‐oriented, diet, exercise, and toxic ED videos than controls. Additionally, participants with increased ED symptoms had TikTok algorithms that were more skewed to these potentially harmful videos (Griffiths et al. 2024). Supporting this, Dondzilo et al. (2024) found that higher engagement with appearance/eating‐related content was associated with increased exposure to that content, which related to greater ED symptoms through increased upward social media comparisons. ED symptoms were also higher among participants who reported that TikTok use negatively influenced their body image (Dahlgren et al. 2024). Two studies with patients with EDs illustrate that TikTok can be both a supportive tool, providing pro‐recovery content, and potentially harmful by feeding pro‐ana content that may inspire users to eat less (Pruccoli et al. 2022; Sjöström et al. 2024).

### 3.3 Experimental Studies

Twenty‐two studies, reported in 17 articles, used experimental and quasi‐experimental designs to test the impact of TikTok content on either (1) EDs, (2) body image/body (dis)satisfaction, or (3) both. Two articles tested the impact of TikTok content on ED symptoms. Both found that participants exposed to eating‐related videos (e.g., #WhatIEatInADay) showed increased negative outcomes ranging from increased shape/weight preoccupation to urges to vomit (Davis et al. 2025; Strickland et al. 2025). One of them reported an experiment with follow‐up data on ED symptoms at 9‐week follow‐up, suggesting that baseline disordered eating levels at baseline predicted greater engagement with restrictive eating content on TikTok at follow‐up (Strickland et al. 2025).

Eleven articles tested the impact of TikTok content on body image/body (dis)satisfaction and related constructs (de Brabandere et al. 2025; Di Michele et al. 2023; Dhadly et al. 2023; Gurtala and Fardouly 2023; Joiner et al. 2023; Kilby and Mickelson 2025; Li et al. 2025; Limniou et al. 2025; Pryde and Prichard 2022; Seekis and Lawrence 2023; Seekis and Lawrence 2024). Female participants exposed to appearance‐relevant content had higher body‐related envy through upward social comparison (Li et al. 2025). Body satisfaction was lower after exposure to “fitspiration,” “posing videos,” thin ideal content, workout videos, or thin dancers' videos than control videos (de Brabandere et al. 2025; Gurtala and Fardouly 2023; Joiner et al. 2023; Limniou et al. 2025; Pryde and Prichard 2022). Participants' lower body satisfaction/increased body dissatisfaction was related to greater appearance comparison (de Brabandere et al. 2025; Pryde and Prichard 2022), upward comparison (Joiner et al. 2023), negative mood (Pryde and Prichard 2022), and self‐objectification (Gurtala and Fardouly 2023).

Five articles found potential benefits of body neutrality/body positivity (Dhadly et al. 2023; Di Michele et al. 2023; Kilby and Mickelson 2025; Seekis and Lawrence 2023; Seekis and Lawrence 2024). Individuals exposed to body neutrality/body positivity content had greater functionality appreciation, body satisfaction, positive thoughts, self‐compassion and self‐esteem, and lower negative affect and self‐objectification. According to Di Michele et al. (2023), body positivity content appears to be a “safeguard” for the effects of exposure to sexualized body ideals, such that participants showed greater body satisfaction after exposure to sexualized body positive content compared with those who were only exposed to the sexualized beauty ideal content. Some studies found slightly different results between exposure to body neutrality versus body positivity content (e.g., Kilby and Mickelson 2025) and others found beneficial effects even among participants in the control conditions (e.g., Dhadly et al. 2023). Overall, both body neutrality and body positivity content showed potential positive effects across the studies in which they were examined, with none of them suggesting detrimental consequences post‐exposure.

Four studies examined the effects of exposure to TikTok both on EDs and body image/body (dis)satisfaction (Blackburn and Hogg 2024; Drivas et al. 2024; Fiuza and Rodgers 2023; Galway and Gammage 2025) and found exposure to pro‐ana content, diet culture content, or #WhatIEatInADay videos led to decreased body satisfaction, increased internalization of appearance ideals, lower positive mood, greater upward social comparison, higher body‐related envy, increased restrictive eating, and intention to diet. Interestingly, Galway and Gammage (2025) did not find differences in any outcomes (i.e., body‐related shame, guilt, envy, and intentions to change diet) between those who were exposed to #WhatIEatInADay videos *with calories* vs. those who did not.

## 4 Discussion

This rapid review identified articles that ranged from content analyses, to observational studies, and quasi‐experimental and experimental designs. There are limitations in the identified articles. Most were cross‐sectional, with the exception of two longitudinal studies (Maes and Vandenbosch 2022; Strickland et al. 2025). Overall, observational and experimental studies recruited White, female (e.g., Primo‐Simões et al. 2025; Seekis and Lawrence 2023) college age students (e.g., Caravelli et al. 2025; Davis et al. 2025), which increases the likelihood of sampling bias and thus may lead to results' unrepresentativeness (Daniel et al. 2024). Further, most of the videos analyzed in content analyses depicted White, female adolescents and young adults, with minor exceptions (Minadeo and Pope 2022).

A strength of our approach was that we restricted inclusion criteria to only studies that examined TikTok specifically, separate from other social media platforms, allowing us to isolate potentially unique effects of TikTok. Studies reporting content analyses illustrate that there is frequent negative‐valenced content that may promote objectification, weight bias, and thin‐ideal internalization (Minadeo and Pope 2022; Munro et al. 2024; Raiter et al. 2023). Recovery‐oriented content is present (e.g., Greene and Norling 2023), although these videos may contain contradictory and/or potentially harmful information as well. Observational studies present concerning results showing that TikTok use is typically associated with increased body image concerns (e.g., Caravelli et al. 2025; Dajches et al. 2024; Mink and Szymanski 2022) and disordered eating (e.g., Dondzilo et al. 2024; Griffiths et al. 2024). However, the results remain mixed, and the lack of conclusive evidence may be attributable to this still being an emerging area of research, and methodological limitations in the existing research. Notably, the potential for “echo chamber” effects, wherein users view content that conforms to and reinforces existing attitudes and beliefs, could be particularly harmful in this domain, as illustrated by the results of Griffiths et al. (2024). Finally, experimental research has begun to demonstrate specific impacts that can result from viewing certain types of TikTok videos, highlighting the importance of better measuring the effects of specific content. Whereas some types of videos (e.g., “fitspiration,” dancing, and eating‐related) may be associated with increased body dissatisfaction (e.g., de Brabandere et al. 2025; Joiner et al. 2023) and/or risk for disordered eating (Blackburn and Hogg 2024; Davis et al. 2025), viewing body neutrality videos may increase self‐compassion (Seekis and Lawrence 2024), with potential positive downstream effects on body image and ED behaviors (Dhadly et al. 2023; Di Michele et al. 2023; Kilby and Mickelson 2025; Seekis and Lawrence 2023; Seekis and Lawrence 2024). Drawing from these conclusions, we propose directions for research using TikTok to understand, prevent, and treat EDs.

### 4.1 Using TikTok to Understand Eating Disorders

Videos on TikTok can impact ED behaviors and body image either positively or negatively. Research increasingly illustrates that the type of content viewed, rather than the amount of time spent on social media, has a larger role in the ultimate impact it has (e.g., Sanzari et al. 2023), highlighting the need for greater nuance in future research. For example, a recent meta‐analysis indicates that experimental exposure to ideal body appearance content appears to exert greater harm on viewers' body image when viewed via TikTok videos compared to Instagram photos (Zhang et al. 2025). By contrast, another meta‐analysis found no significant association between use of short‐form video social media (frequency, duration, and/or intensity) and body image outcomes (Nguyen et al. 2025). There are a number of factors that may influence the mixed findings reported in our review. Variables such as format (e.g., photo versus video), content (e.g., pro‐anorexia, thin ideal, “fitspiration,” or body neutrality/body positivity), context (i.e., laboratory versus naturalistic use), method of use (i.e., active versus passive), and person‐level differences (e.g., sociodemographic characteristics such as age; media literacy; baseline body image and/or disordered eating) may moderate these relationships. More research is needed to understand what content is helpful versus harmful, who is at risk versus protected, and when. Researchers pursuing these questions should ground their research questions and hypotheses in theoretical models of how sociocultural factors influence EDs, such as the tripartite model (Thompson et al. 1999) or objectification theory (Fredrickson and Roberts 1997). Experimental methods may be particularly well suited to initial tests of possible moderators. To support research with this approach, one research team developed “EDTok,” a database of over 43,000 TikTok videos with ED‐related content (Bickham et al. 2025). This tool will allow researchers to leverage experimental designs to test various impacts of exposure to TikTok using standardized experimental stimuli.

Proportionally more research has focused on body image rather than disordered eating, indicating an empirical gap that can be met by ED researchers. The results from Griffiths et al. (2024) illustrate a dangerous synergy between the content pushed by the algorithm and attentional biases in users with EDs. Moreover, these authors suggest that the algorithm appears to be more responsive to passive user behaviors (e.g., viewing time) and desensitized to active actions (e.g., liking videos). This appears to result in TikTok users with EDs being exposed to videos that could exacerbate their ED behaviors, a finding that is consistent with other studies in patients with EDs, both qualitative (Sjöström et al. 2024) and quantitative (Pruccoli et al. 2022). Future research on TikTok and EDs should include both experimental research, like that described here, and longitudinal designs that allow for testing of within‐person effects. Moreover, the research is biased to young, mostly White women and non‐clinical samples; researchers must recruit diverse samples more representative of both TikTok's user base (Gu et al. 2022) and people with EDs (Assari and DeFreitas 2018; Simone et al. 2022).

The study by Griffiths et al. (2024) is unique in its approach of exploiting the individual data of users. Although early research indicates what content is present on TikTok and how certain types of content *can* impact individuals, these methods have largely relied on navigating *around* the hyper‐personalized nature of users' algorithmically‐determined TikTok feeds, rather than capitalizing on this feature of the platform. These researchers obtained longitudinal (i.e., over one month) user data, downloaded from the app by participants themselves, to observe naturalistic use of the app. Other researchers have implemented a “data donation system” in which users can request their data from the app, per their right of access as delineated in the European Union's General Data Protection Regulation (Council of the European Union 2016), and then anonymize their data prior to providing them to researchers (Zannettou et al. 2024). These forms of data collection, which provide longitudinal, detailed, and highly individual data, may make it possible to examine not only how TikTok can impact individuals' body image, emotions, and behaviors in controlled research settings, but also what real‐world impact TikTok has on users over time. Collecting such data from participants new to using TikTok might expose how the naive algorithm responds to new users over time, responding to their usage patterns and shaping their experience with the app. If combined with methods such as ecological momentary assessment (Shiffman et al. 2008) and/or passive forms of data collection (Levinson et al. 2019), these data may make it possible to develop rich models of how TikTok use and content may relate to ED behaviors in real time. Furthermore, researchers could conduct mixed‐methods research to better understand what type of content users are hoping to find on TikTok (e.g., community, advice for achieving recovery), whether they perceive their FYP to meet those interests, and whether the videos fed by the algorithm are likely to meet those needs and desires. Research along these lines is likely to further pry open the “black box” of the TikTok algorithm, which may help to identify which individuals are at most risk of experiencing the potential negative impacts of TikTok that have been demonstrated in experimental studies. At the same time, researchers must consider the ethical issues raised by having access to such detailed data and how such matters interact with efforts toward providing open access to data (Kanthawala et al. 2022; Zannettou et al. 2023). Finally, it remains to be seen what effects described here are general to short‐form video across platforms and which are specific to TikTok, which would point to the role of the platform's unique algorithm. Most studies have attempted to answer the question of whether eating‐related short‐form videos in social media platforms impact EDs. For instance, Gurtala and Fardouly (2023) provided participants with stimulus materials without the Instagram or TikTok format (i.e., no Instagram or TikTok borders), and they found that results were applicable to a wide range of social media platforms. Recently, researchers have started to address the unique TikTok algorithm question (see Griffiths et al. 2024). There is value in understanding both the specific dangers of the TikTok algorithm and what impacts may result from other short‐form video platforms.

### 4.2 Using TikTok to Prevent and Treat Eating Disorders

While much of the research to date has focused on negative impacts, we encourage researchers to consider positive impacts that TikTok could have, including the potential to prevent ED onset and/or support recovery. Experimental studies have found positive effects of body‐neutrality and body‐posivity TikTok content on body satisfaction and mood (Dhadly et al. 2023; Seekis and Lawrence 2023), suggesting that these forms of content may serve as a “buffer” or protective factor regardless of the TikTok content that individuals are exposed to (Di Michele et al. 2023), and could therefore serve as a form of ED prevention. Given the importance of social media in adolescents' lives, TikTok may be an ideal platform to disseminate evidence‐based ED prevention and brief interventions. According to a study by Gallup, children and adolescents spend nearly 5 h online daily and 1.5 h on TikTok specifically (Rothwell 2023). They frequently seek mental health information on social media but may encounter misinformation and/or harmful information. A recent field experiment found that mental health content creators on TikTok could be trained to provide evidence‐based mental health content to their followers (Motta et al. 2024), and influencers could be partners in providing body image interventions (Mahon and Hevey 2023; Paraskeva et al. 2024). Targeting misinformation with empirically‐supported strategies such as social media literacy training shows promise (Georgiou et al. 2025; Polanco‐Levicán and Salvo‐Garrido 2022). Such an approach would be consistent with harm‐reduction principles, which encourage a shift in behavior rather than withdrawal of an entire behavior (i.e., instead of eliminating drugs, harm reduction promotes ensuring injections are done safely; Marlatt 1996). Similarly, with TikTok and other social media, it is crucial to meet users where they are. Asking individuals to stop using TikTok due to potential or real risk of harm is unrealistic, and asking people to stop turning to social media for community is unlikely to succeed (Xu et al. 2023). Working to instead leverage these platforms and the communities that use them is likely to have greater reach and larger benefit.

Finally, TikTok may hold potential as a tool in the ED clinician's toolbox. In addition to using TikTok to recruit participants for clinical trials (Lee et al. 2024), researchers and clinicians should consider novel ways to incorporate TikTok into treatment. Although some studies used content analysis to focus on the ED recovery community on TikTok (e.g., Greene and Norling 2023; Herrick et al. 2021; Kells et al. 2025), there were no experimental studies, leaving an opportunity for research in this area. Providing patients with tools to access and effectively use the ED recovery community on TikTok as part of recovery could supplement gains made in treatment. For instance, clinicians could provide patients a “tips and tricks” guide on getting involved in TikTok recovery communities, and how to navigate away from potentially harmful content. Moreover, although EDs have significantly elevated comorbidity and mortality rates (Krug et al. 2025), only about 20% of those with EDs receive an accurate diagnosis, and even fewer receive appropriate care due to treatment barriers (Hach et al. 2005; Penwell et al. 2024) and early dropout (Martin‐Wagar et al. 2021). To our knowledge, no studies have explored the possibility of disseminating psychoeducation and/or interventions for EDs through TikTok. Single session interventions (SSIs) have the potential to increase the accessibility, scalability, and cost‐effectiveness of mental health services (Schleider and Weisz 2017). SSIs intentionally involve only one encounter with a program or provider (Schleider et al. 2023). By using TikTok, a platform where users (especially adolescents and young adults) already spend time and feel comfortable, researchers might be able to capture youths' attention and ensure high engagement following SSI principles. Targets that may be amenable to treatment via SSI include thin ideal internalization (Becker et al. 2017), diet culture or fat talk (Shannon and Mills 2015), social media literacy (Cho et al. 2024), and beliefs/awareness of peer norms (Forney and Ward 2013). Advances in using TikTok for prevention and treatment of EDs will unfold in conversation with research examining the role of TikTok in EDs, as scholars develop new understanding of how this unique form of social media influences disordered eating and body image.

## 5 Conclusions

This rapid review demonstrates potentially harmful effects of TikTok use for body image and disordered eating, along with possible benefits of body‐positive or body‐neutral content on social media. Still, there are multiple open questions regarding TikTok and EDs, making this a promising and exciting area for research. By using innovative methods, we may gain new insights into the development, phenomenology, prevention, and treatment of EDs. The time to seize this opportunity is now!

## Author Contributions

**Macarena Kruger:** conceptualization, project administration, validation, writing – review and editing, writing – original draft. **Isabella Pruscino:** conceptualization, writing – original draft, writing – review and editing. **Caroline G. Martin:** conceptualization, writing – original draft, writing – review and editing. **Elizabeth A. Velkoff:** conceptualization, supervision, writing – original draft, writing – review and editing.

## Lived Experience Involvement Statement

No specific efforts were undertaken to involve persons with lived experience in the study design or execution, or in the preparation of this manuscript.

## Funding

The authors have nothing to report.

## Conflicts of Interest

The authors declare no conflicts of interest.

## Data Availability

Data sharing not applicable to this article as no datasets were generated or analyzed during the current study.
